# Intermolecular Hydrogen-Bonded Interactions of Oxalic Acid Conformers with Sulfuric Acid and Ammonia

**DOI:** 10.1021/acsomega.4c06290

**Published:** 2024-10-01

**Authors:** Eduardo da Silva Carvalho, Angsula Ghosh, Puspitapallab Chaudhuri

**Affiliations:** Department of Materials Physics, Federal University of Amazonas, Manaus, AM 69080-900, Brazil

## Abstract

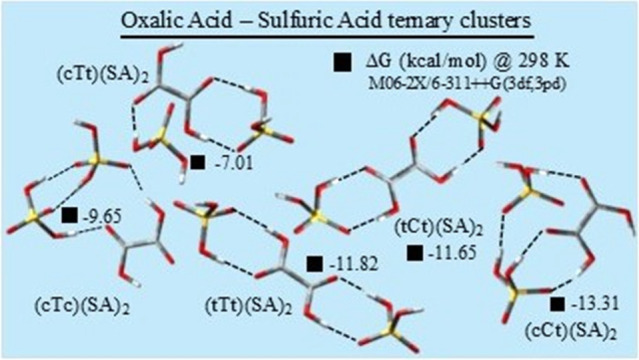

Oxalic acid is one of the simplest naturally occurring dicarboxylic acids that is abundantly found in the atmosphere, and it has several stable structural conformers. Hydrogen-bonded interactions of oxalic acid with other atmospheric molecules are important, as they might influence the chemical composition of the atmosphere, thereby impacting atmospheric chemistry and environmental processes. In this work, we used density functional calculations with the M06–2*X*/6-311++G(3df,3pd) model to examine the interaction of five oxalic acid conformers with sulfuric acid and ammonia—two widely recognized atmospheric nucleation precursor molecules—with the aim of observing the hydrogen-bonding characteristics of the conformers individually. An extensive and systematic quantum-chemical calculation has been conducted to analyze the structural, thermodynamical, electrical, and spectroscopic characteristics of several binary and ternary clusters mediated by five oxalic acid conformers. Our analysis of the electronic-binding energies and free energy changes associated with the formation of the clusters at ambient temperature reveals that multiple conformations of oxalic acid have the potential to engage in stable cluster formation in the atmosphere. In fact, the highest energy oxalic acid conformer exhibits the lowest bonding free energy in most cases. According to our calculations, clusters of oxalic acid with sulfuric acid demonstrate greater thermodynamic stability, a higher probability of formation, and more intense light scattering compared to clusters with ammonia. Furthermore, the analysis of successive cluster formation reveals that clusters formed between sulfuric and oxalic acids are more likely to grow spontaneously than those formed between ammonia and oxalic acid.

## 1 Introduction

Hydrogen-bonding interactions among organic molecules are of great importance in many different branches of chemical sciences such as biochemistry and molecular biology, medicinal chemistry and drug designing, materials science/polymer chemistry, astrochemistry, atmospheric chemistry, and so on. The nature of the hydrogen bonds (HBs) may directly influence the stability of molecular structures, reactivities, and functions. In living organisms, HBs are fundamental in maintaining the secondary and tertiary structures of biomolecules such as proteins and nucleic acids. Understanding HBs is crucial in materials science for designing and manipulating the properties of polymers, supramolecular assemblies, and other advanced materials. In the atmosphere, organic compounds, such as carboxylic acids, can interact with other molecules via hydrogen bonding to form small stable clusters. These clusters can grow in size and eventually lead to the formation of secondary organic aerosols. Oxalic acid (OA, C_2_H_2_O_4_) is one such molecule of significant atmospheric importance.^1−24^ It is the simplest naturally occurring water-soluble dicarboxylic acid (DCA) found in various environmental sources, such as plants, fungi, and some marine organisms.^1,20,25−27^ In human body, OA is produced through the metabolism of certain plant-based foods like leafy greens (such as spinach, rhubarb, amaranth), nuts, seeds, tea, sweet potatoes, okra, etc.^28−31^ In the atmosphere, it may be formed via oxidation of larger compounds such as isoprene and monoterpenes, volatile organic compounds emitted by transpiration from plant leaves.^32−34^ DCAs, in general, are common organics identified both in the urban and rural areas mainly due to the intense agricultural and industrial activities and are an important constituent of the nucleation process due to their low vapor-pressure.^19,21−23,35−42^ Moreover, being water-soluble, DCAs may serve as cloud condensation nuclei, becoming relevant in the global climate system. OA is reported to be the most-abundant atmospheric DCA detected in the air as a major constituent of ultrafine and fine aerosol particles.^8,21−23,38,43−45^ Notably, the gas-phase concentration of OA is reported to be in the range of 9.3 × 10^10^ to 5.4 × 10^12^ molecules/cm^3^,^23,24,45^ which is much higher than that of ammonia (AM) and sulfuric acid (SA), as the typical concentrations of SA and AM are in the range of 10^4^–10^8^ molecules/cm^3^ and 10^7^–10^11^ molecules/cm^3^, respectively.^5,16,23,24,46,47^ OA and SA are two crucial precursor molecules in atmospheric nucleation, contributing significantly to the formation of secondary aerosols.^48−55^ The molecular configuration of OA, with two carboxylic (COOH) groups on either side of a C–C bond, imparts substantial rotational flexibility, leading to the possible existence of several conformers, each with distinct structural arrangements. Over the past few decades, several experimental^56−61^ and theoretical^21,60−69^ investigations explored the intricate conformational landscape of OA. Theoretical predictions suggest that in the gas phase, OA should have at least five different structural conformations. The three lowest energy conformations predicted by theory have been observed experimentally.^59−61^ On the other hand, the presence of two COOH groups enables OA to engage in a greater number of hydrogen bonding interactions, compared to monocarboxylic acids, with two carbonyl (C=O) groups acting as proton acceptors and the OH groups acting as proton donors. Recent theoretical investigations on atmospheric nucleation and new particle formation reveal that OA can generate thermodynamically stable hydrogen-bonded clusters with atmospherically relevant molecules such as SA, water, AM, amines, and methanesulfonic acid.^3−8,12−19,21,23,24^ While the contribution of hydrogen-bonded interactions of oxalic acid, particularly considering its lowest energy conformations or a few others, in atmospheric aerosol formation has been widely studied, the hydrogen-bonding characteristics of individual oxalic acid conformers have not yet been fully explored. Quantum chemical calculations on the binary and ternary clusters OA with AM and H_2_O,^5,6^ hydration of OA dimer,^15^ OA-catalyzed hydration reaction of SO_3_,^21^ and dissociation of oxalic acid in water clusters^67^ are the few ones that considered all the five OA conformers. In the present work, we consider the effect of hydrogen-bonded interactions of five stable OA conformers with atmospheric molecules such as SA and AM. A detailed quantum-chemical analysis has been conducted, and relevant structural, thermodynamical, and spectroscopic properties of multiple conformations of various binary and ternary clusters are reported.

## 2 Computational Methods

The molecular geometries of OA, SA, and AM and those of the hydrogen-bonded binary and ternary clusters formed with these molecules were fully optimized without any constraint in the gas phase. The hybrid DFT functional M06–2X, which is parametrized to account for dispersion interactions, was used for this purpose with Pople’s split-valence triple-ζ 6–311++G(3df,3pd) basis set, maintaining consistency with our recent work on organic atmospheric molecules.^70,71^ This M06–2*X*/6-311++G(3df,3pd) model is well recommended in the literature for quantum-chemical analysis of hydrogen-bonding interactions among atmospheric molecules.^50,72−75^ Five structurally different conformers of OA monomer have been considered whose initial geometries were prepared based on previous works.^21,60−69^ The initial structure clusters were then prepared following a multistep approach, considering the fact that all the monomers (OA, SA, and AM) can simultaneously act as proton-acceptor and proton-donor, facilitating the formation of multiple HBs. Moreover, depending on the relative position of the COOH groups on either side of the central C–C bond, different forms of intermolecular arrangement for hydrogen bonding are possible. As the main objective of this work is to observe how different conformations of OA influence its hydrogen-bonding characteristics, we searched for different possible HB patterns for each OA conformation. Initially, several binary or ternary clusters were prepared by strategically placing SA and/or AM around the hydrogen-bonding sites of OA using the GaussView molecular visualization program,^76^ and all of them were optimized using the M06–2*X*/6-31++G(d,p) model. Some of these optimized structures were then selected based on energy and structural distinctness, and further optimized by the larger M06–2*X*/6-311++G(3df,3pd) model. Finally, a few of the lower energy cluster geometries for each OA monomer were chosen for the final analysis. After each geometry optimization, the vibrational frequencies were obtained at the same level of calculation to ensure that all frequencies are positive and that the optimized geometry is a local minimum on the potential energy hypersurface.

Thus, in this work, we consider each of the five OA conformers individually interacting with AM and SA to form the (OA)(AM) and (OA)(SA) dimers, as well as the (OA)(AM)_2_, (OA)(SA)_2_ trimers. This results in a total of 10 different dimer compositions and 10 different trimer compositions. Each cluster composition, on the other hand, contains multiple structural conformations with varying electronic energies in most cases, as we discuss in the next section.

The binding electronic energy (Δ*E*) and the binding Gibbs free energy of formation (Δ*G*) were calculated for each cluster considering the usual supermolecular approach:
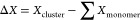
1where *X = E* (electronic energy of the system) or *G* (electronic energy with thermal free energy correction). Both *E* and *G* are corrected for the zero-point energy (ZPE). As each cluster composition may possess several energetically stable conformers, the effect of multiple conformers on the cluster-binding free energy is calculated as,^50,77,78^
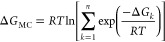
2where *R =* 8.314J/(mol K) is the universal gas constant and *T* is the ambient temperature (298.15 K).

When molecules come together to form a cluster or complex, they may undergo changes in their geometries to adapt to a new environment or interactions. The energy required for these structural adjustments is commonly termed the distortion energy or relaxation energy. In mathematical terms, the distortion energy of a molecule engaged in clustering is determined by subtracting the electronic energy of that molecule (monomer *i*) in its isolated form (*E*_*i*_) from the energy of the same monomer with its geometry altered and fixed within the cluster (). The total distortion energy of the cluster containing *N* monomers, Δ*E*_D_ (tot), is the sum of the individual distortion energies of all monomers within the cluster. Thus,
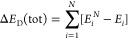
3

In physical terms, Δ*E*_D_, a positive quantity, is a measure of the strain or perturbation introduced into the system due to the molecular clustering process.

Since the probability of a given set of molecules arranging themselves in a particular configuration *k* with Gibbs free energy change of Δ*G*_*k*_ is proportional to the Boltzmann factor of the cluster formation, exp(−Δ*G*_*k*_/*RT*), the relative population fraction, RPF(*k*) of different conformations in a particular cluster composition were calculated by using the following relation:
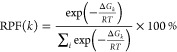
4

The optical properties, such as the Rayleigh scattering intensities () and depolarization ratios (σ) for natural light, of the monomers and OA-mediated clusters were obtained, at the same M06–2*X*/6-311++G(3df,3pd) level, using the following definitions.^79−82^
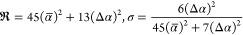
5where,  and Δα are the mean isotropic polarizability and the anisotropy of polarizability of the molecular system,
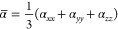

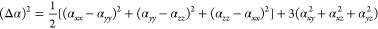
6

All calculations were performed using the Gaussian 16^83^ computational chemistry software package. Preparation of the initial molecular structures and partial analysis of the calculated results were done by the GaussView interface.^76^

## 3 Results and Discussion

### 3.1 Isolated Molecule of Oxalic Acid

**Figure 1:**
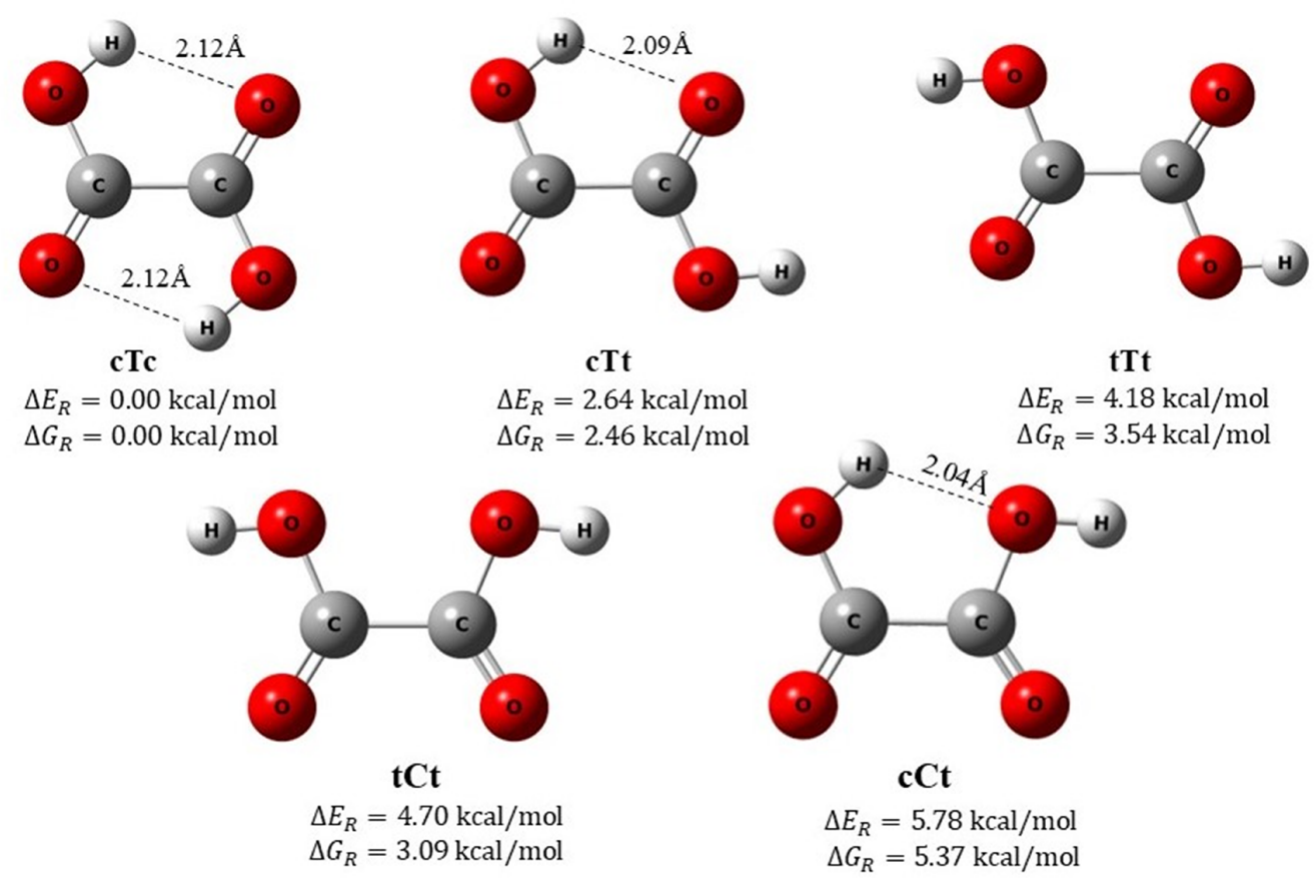
Structures of the five stable oxalic acid (OA) conformers, optimized using the M06–2*X*/6-311++G(3df,3pd) model, showing intramolecular hydrogen bonds (dotted lines) along with the relative electronic energy (Δ*E*_R_) and relative Gibbs free energy (Δ*G*_R_), calculated with respect to the lowest energy conformer, cTc.

The geometries of the five oxalic acid (OA) conformers, optimized by the M06–2*X*/6-311++G (3df,3pd) model, are illustrated in Figure 1. The structures are arranged in order of increasing relative electronic energy (Δ*E*_R_), with zero-point energy corrections applied, calculated relative to the conformer with the lowest electronic energy. The conformers are named hereafter as cTc, cTt, tTt, tCt, and cCt following the literature.^5,21,59,60,62,66,67^ The nomenclature is based on the torsional degrees of freedom of the OA structures. The uppercase letters C and T represent the cis and trans configurations, respectively, of the O=C–C=O dihedral angle corresponding to the internal rotation of the carboxyl COOH groups around the C–C bond. The lower case letters c and t, on the other hand, signify the cis and trans configurations of the two C–C–O–H dihedral angles, representing the rotation of the OH group about the C–O bond in each COOH group.

In the lowest energy conformation of OA (cTc), two carboxyl groups are trans to each other, i.e., φ(0=C–C=0) = 180°, and both OH groups are cis with respect to the C–C bond. As a result, cTc is stabilized by the formation of two intramolecular O–H···O HBs of equal bond length (2.12 Å) and bond angle (115.5°). The second-most stable conformation (cTt) differs from cTc by the internal rotation of one of the two OH groups about the C–O bond, making it trans with respect to the C–C bond. As a result, cTt possesses only one intramolecular HB, which has a bond length of 2.09 Å. The energy difference between cTc and cTt is 2.68 kcal/mol which matches with 2.62 and 2.75 kcal/mol, obtained by higher level energy calculations with MP2/aug-cc-pVDZ and CCSD(T)/aug-cc-pVTZ//B3LYP/6-311++G(d,p) models, respectively.^5^ Other relative energies from MP2 (CCSD) calculation,^5^ are Δ*E*_R_(tTt) = 3.89 (4.31) kcal/mol, Δ*E*_R_(tCt) = 4.43 (4.99) kcal/mol and Δ*E*_R_(cCt) = 5.69 (6.02) kcal/mol. As can be observed from Figure 1, these values are also in good agreement with those obtained by the M06-2X calculation. The cTc, cTt, and tTt are the only conformers that have been detected by experiments.^59−61^

The calculated values of the structural parameters like bond lengths and bond angles of the OA conformers agree well with experiments and previous calculations.^62,66^ Since, detailed discussions on each of these OA conformer structures are available in several previous works on OA, we provide the structural parameters obtained by our M06–2*X*/6-311++G(3df,3pd) along with others from the literature in Table S2. Here, we discuss briefly some of the electric and spectroscopic parameters reported in Table 1, which are also important for characterization of the conformers.

**Table 1:** Calculated Values of Dipole Moment (μ), Mean Dipole Polarizabilty (), Polarizability Anisotropy (Δα), Rotational Constants (*A*, *B*, and *C*)^a^, Degree of Depolarization (σ), and Rayleigh Activity () for Natural Light of the Five OA Conformers as Obtained by M06–2*X*/6-311++G(3df, 3pd) Model

	cTc	cTt	tTt	tCt	cCt
μ (D)	0.00	3.15	0.00	2.98	4.89
α (a.u.)	37.36	37.84	38.13	38.16	37.99
Δα (a.u.)	20.37	19.72	19.53	19.60	19.72
*A* (GHz)	5.878	6.027	6.149	6.120	6.000
*B* (GHz)	3.855	3.708	3.611	3.619	3.672
*C* (GHz)	2.328	2.296	2.275	2.274	2.278
σ (a.u.)	0.044	0.041	0.039	0.039	0.040
(a.u.)	68188.7	67140.3	68103.4	68198.4	69983.7

a Experimental values of the rotational constants (GHz): *A* = 5.951, *B* = 3.684, *C* = 2.276.^61^.

As can be seen from Table 1, cTc and tTt are the only OA conformers among the five that have zero dipole moment, as a consequence of their structural feature. Both possess C_2h_ symmetry, with the two COOH groups having a trans configuration with respect to each other. So, the dipole moment vectors corresponding to each group acts in opposite direction nullifying the total dipole of the system. The dipole moment of 3.15D for cTt, calculated by the present M06–2*X*/6-311++G(3df, 3pd), agrees well with the experimentally measured value of 3.073(6)D^61^ and B3LYP/aug-cc-pVDZ calculated value of 3.14D.^5^ Apart from cTc and cTt, the highest energy conformer cCt also has one intramolecular HB. Curiously, it has the shortest HB length (2.12 Å) and highest dipole moment (4.89 D) among all the clusters. The same observation was also found in B3LYP/6-311++G(d,p) calculation.^5^ Since both the OH groups in the lowest energy cTc conformer are oriented inwardly, it has the lowest molecular volume with an electronic spatial extent (ESE) of 479 au. On the other hand, both the tTt and tCt conformers with their OH groups looking outward in opposite directions have the highest volume with ESE ≈ 491 au. ESE is a measure of the average size of the electron distribution in a molecule. Molecules with a larger ESE have more diffuse electron clouds, which generally leads to greater polarizability, as the extended electron cloud can be more easily distorted by an external field. As a result, tTt and tCt have the highest mean dipole polarizabilities with α = 38.13 au and 38.16 au, respectively, while cTc has the lowest polarizability (α = 37.36 au). However, in general, the difference in polarizability between the conformers is minimum. In case, of degree of depolarization (σ) all the conformers have the same and a very small value with *σ ≈* 0.04 au which signifies that the polarization state of the incident light will remain almost unaltered during the scattering process with OA conformers. As far as the elastic light scattering (Rayleigh scattering) is concerned, the scattering intensities also do not vary appreciably from one conformer to another, but the highest energy cCt shows the highest Rayleigh activity, suggesting most effective scattering of incident radiation by this conformer.

**Figure 2:**
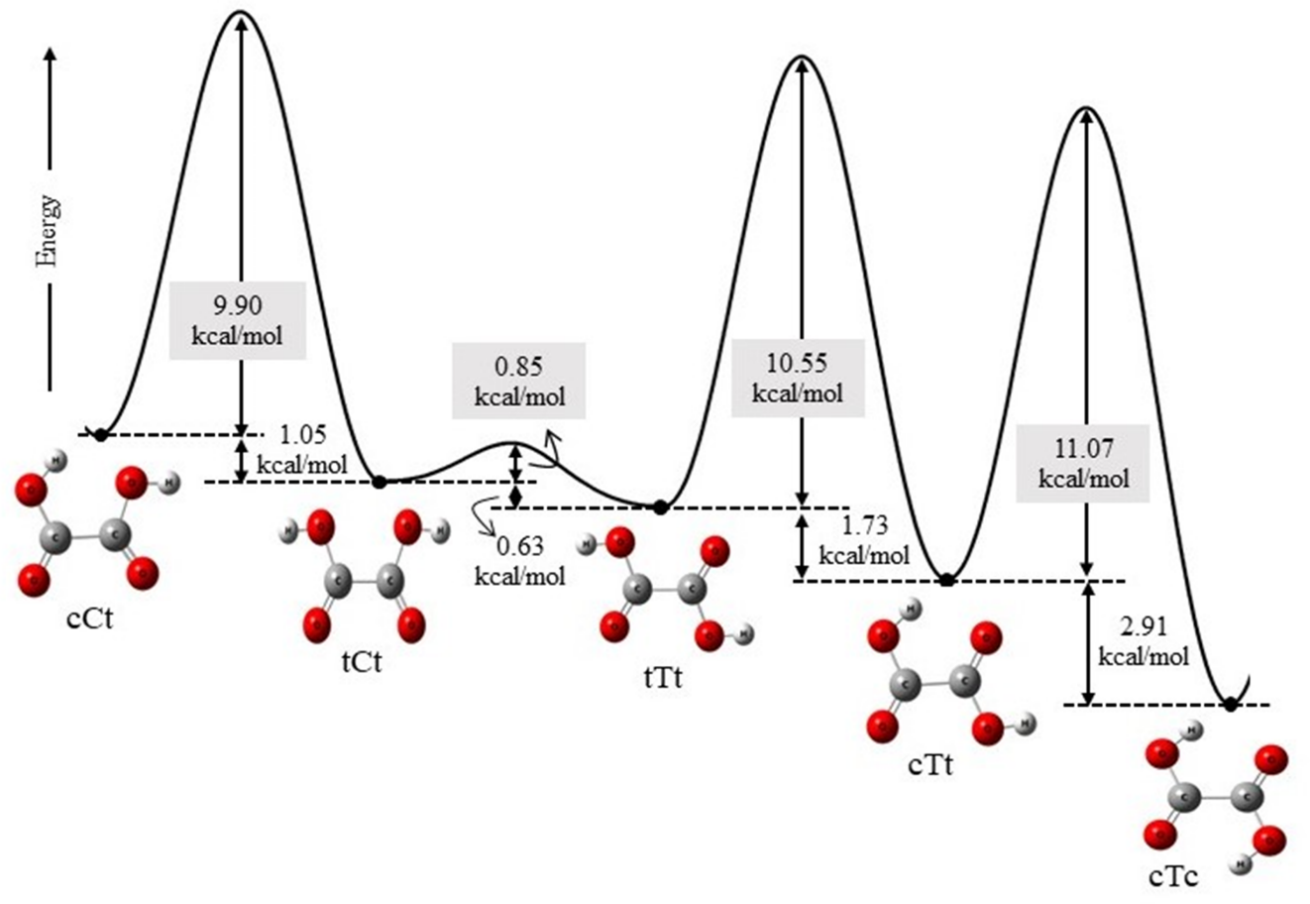
Schematic diagram of the rotational barriers for the possible interconversions among the OA conformers as obtained by the M06–2*X*/6-311++G(3df, 3pd) level.

A sequential interconversion among the conformations in either decreasing or increasing order of electronic energy (cCt ⇄ tCt ⇄ tTt ⇄ cTt ⇄ cTc) is possible just by rotation of a single dihedral angle in each confirmation. Figure 2 illustrates a schematic representation of the rotational energy barriers for these interconversions, obtained through the constrained optimizations (energy scan) of the molecule, rotating a selected dihedral angle in small increments of 3° and optimizing the molecular geometry while keeping the dihedral angle fixed at the incremented value. For instance, rotating a COOH group about the central C–C bond or the C–C–O–H dihedron allows the transition from the tCt to the tTt conformer. The rotational energy barrier in this case is remarkably low, measuring just 0.86 kcal/mol for tCt → tTt and 1.48 kcal/mol for tTt → tCt, representing the minimum barrier encountered by our calculation at the M06–2*X*/6-311++G(3df, 3pd) level. The highest energy barriers are observed for the cTc → cTt and cTt → cTc interconversions, with values of 13.98 and 11.07 kcal/mol, respectively. These transitions involve the rotation of a specific O—C–O–H dihedral, transitioning from a trans to cis configuration. The calculated barriers agree well with previous predictions with different models.^62,63^ As can be observed in the figure, with the exception of tCt ⇄ tTt, the rotational barriers are significantly high, suggesting that the cCt, cTt, and cTc conformers possess considerable stability under normal conditions.

Among the five OA conformers, cTc, tTt, and tCt contain degenerated vibrational stretching modes for the OH group as a consequence of the symmetry of the molecule. In the cTc conformer, the two equivalent OH groups asymmetrically stretch at 3727 cm^–1^ with an intensity of 306.6 km/mol, while they symmetrically stretch at 3723 cm^–1^ with an almost negligible intensity of 0.012 km/mol. For both tTt and tCt conformers, the vibrational frequencies for these coupled symmetric and asymmetric stretching modes are practically the same, measuring 3832 cm^–1^ for tTt and 3821 cm^–1^ for tCt. However, the intensity of the asymmetric vibration is significantly higher than the symmetric vibration in both cases. In contrast, for cTt and cCt conformers, the OH groups exhibit independent stretching modes with similar intensities of vibration. The calculated OH stretching frequencies for the cis-CCOH (trans-CCOH) of cTt and cCt are approximately 3773 (3819) cm^–1^ and 3824 (3830) cm^–1^, respectively. Considering the experimentally observed OH stretching frequencies in the range 3453–3461 cm^–1^, we notice that the M06–2*X*/6-311++G(3df,3pd) level of calculation overestimates the OH stretching frequencies by ca. 7%.

### 3.2 Clusters of Oxalic Acid and Ammonia, (OA)(AM)_*n*_ (*n*= 1,2)

**Figure 3:**
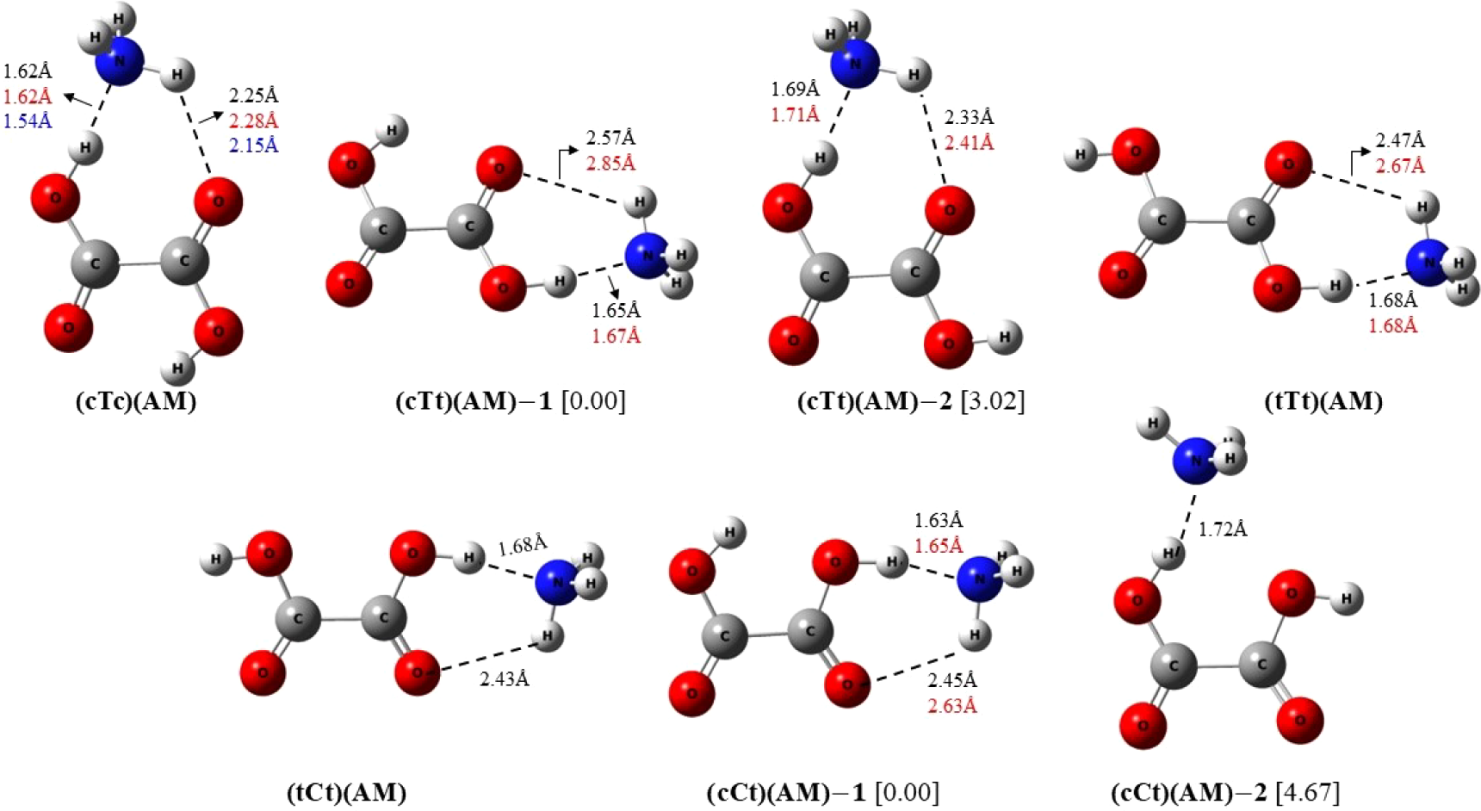
Equilibrium geometries of the stable (OA)(AM) cluster compositions optimized at the M06–2*X*/6-311++G(3df,3pd) level. The dashed lines represent the intermolecular hydrogen bonds with respective bond lengths, obtained using the present model shown in black color, while those from other models—B3LYP/aug-cc-pVDZ^6^ and PW91PW91/6-311++G(3df,3pd)^7^—are shown in red and blue, respectively. The numbers in square brackets represent the relative energy differences of the conformations within each cluster composition in kcal/mol.

**Figure 4:**
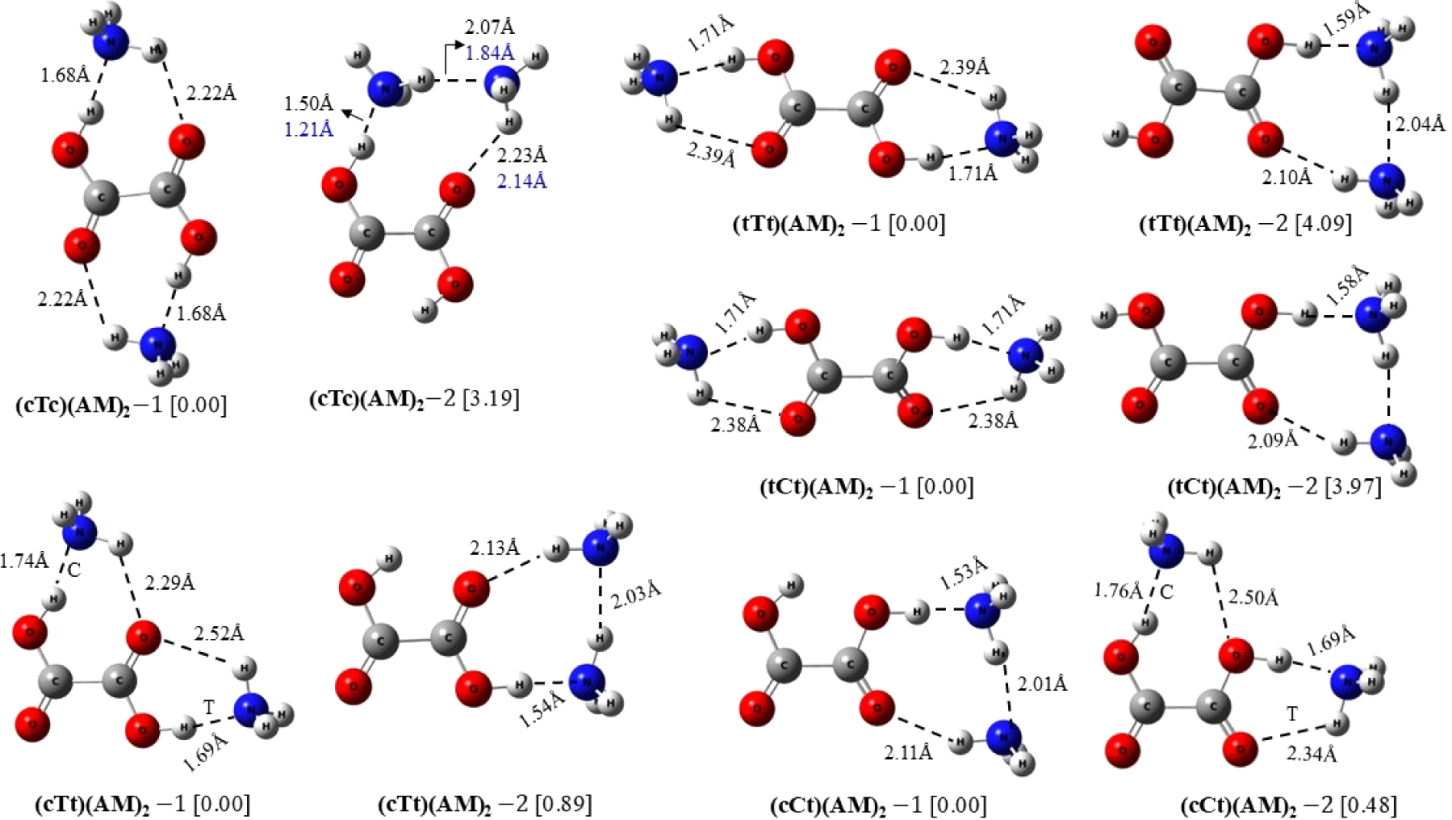
Equilibrium geometries of the stable (OA)(AM)_2_ cluster compositions. optimized at the M06–2*X*/6-311++G(3df,3pd) level. The dashed lines represent the intermolecular hydrogen bonds with respective bond lengths, obtained using the present model shown in black, and those from PW91PW91/6-311++G(3df,3pd)^7^ in blue. The numbers in square brackets represent the relative energy differences of the conformations within each cluster composition in kcal/mol.

In this section, we analyze the structural and thermochemical properties of the binary and ternary clusters formed by one molecule of the five conformers of OA (cTc, cTt, tTt, tCt, cCt) with AM. Figure 3 exhibits the optimized geometries of the seven binary (OA)(AM) clusters, with (cTt)(AM) and (cCt)(AM) compositions having two conformations each, while (cTc)(AM), (tTt)(AM), and (tCt)(AM) have a single conformation because of structural symmetry. Figure 4 displays the optimized structures of 10 ternary (OA)(AM)_2_ clusters, considered for the present work, with each of the five cluster compositions having two structural conformations. Although more than two conformations were identified for certain (OA)(AM)_2_ compositions, we selected the two lowest energy conformations, with distinct structural features, for each ternary composition.

Some of these hydrogen-bonded structures have been previously studied. Specifically, the binary structures (cTc)(AM), (cTt)(AM)-1, (cTt)(AM)-2, (tTt)(AM), and (cCt)(AM)-1 were investigated using the B3LYP/aug-cc-PVDZ model.^6^ Additionally, the binary (cTc)(AM) and ternary (cTc)(AM)_2_-1 structures were studied by the PW91PW91/6-311++G(3df,3pd) model.^7^ The intermolecular hydrogen bond (HB) distances obtained by these models are shown in the figures alongside those obtained by the present model. As evident, the HB distances calculated by the M06–2*X*/6-311++G (3df,3pd) model are in excellent agreement with those of the B3LYP/aug-cc-PVD model, while PW91PW91/6-311++G(3df,3pd) predicts shorter HB distances. The HB interactions occur via the −OH and −CO groups of OA where it acts simultaneously as a proton donor and proton-acceptor, respectively. As the figures show, the HB distances in the possible (N)H···O type interactions between OA and AM, with the former being the proton acceptor, are, in general, much larger than the (O)H···N HB distances when OA acts as proton donor. The HB bond angles are also larger in the case of the (O)H···N bonds. Thus, we assume that the formation of (O)H···N HBs between OA conformers and AM molecules with OA being the proton-donor via its O–H moiety and AM being the proton acceptor via the nitrogen atom is responsible for the energetic stability of the (OA)(AM) clusters. We report in Table 2 some structural and spectroscopic parameters required for the characterization of these (O)H···N HBs like the distance between the two electronegative heavy atoms (oxygen and nitrogen) participating in the HB formation (*R*_O–N_), The HB length (*R*_(O)H···N_), HB angle (∠O–H···N), elongation of the O–H bond due to HB formation (Δ*R*_O–H_), O–H stretching frequency in the clusters and its red-shift with respect to the isolated OA molecule. The Cartesian coordinates of all the optimized geometries of (OA)(AM) and (OA)(AM)_2_ clusters are given in Table S2a,b, respectively.

**Table 2:** Relevant HB Parameters of the Binary (OA)(AM) and Ternary (OA)(AM)_2_ Clusters Including the HB Distance, O–H Bond Length, Variation of the Bond Length, HB Angle, and O–H Frequencies and the Variation of the O–H Frequencies upon Cluster Formation^a^

		*R*_O–N_ (Å)	*R*_(O)H···N_ (Å)	∠O—H···N (degrees)	Δ*R*_O–H_ (Å)	*v*_O–H_ (cm^–1^)	Δ*v*_O–H_ (cm^–1^)
(OA)(AM)							
(cTc)(AM)		2.638	1.619	171.1	0.054	2692	–1035
(cTt)(AM)-1		2.659	1.649	170.7	0.052	2786	–1034
(cTt)(AM)-2		2.685	1.690	167.4	0.040	2974	–799
(tTt)(AM)		2.681	1.682	168.6	0.045	2904	–928
(tCt))(AM)		2.678	1.678	168.2	0.046	2917	–904
(cCt)(AM)-1		2.641	1.632	168.4	0.056	2753	–1071
(cCt)(AM)-2		2.696	1.719	164.1	0.037	3063	–767
(OA)(AM)_2_							
(cTc)(AM)_2_-1		2.678	1.678	168.2	0.042	2894^b^	–833
(cTc)(AM)_2_-2		2.562	1.503	172.1	0.094	2121	–1607
(cTt)(AM)_2_-1	C	2.722	1.740	166.4	0.034	3101	–672
	T	2.692	1.693	170.3	0.041	2977	–843
(cTt)(AM)_2_-2		2.596	1.545	177.7	0.084	2287	–1532
(tTt)(AM)_2_-1		2.704	1.714	167.5	0.039	2997^c^	–835
(tTt)(AM)_2_-2		2.623	1.586	178.2	0.071	2495	–1337
(tCt)(AM)_2_-1		2.701	1.710	167.3	0.040	2985^d^	–836
(tCt)(AM)_2_-2		2.622	1.567	178.7	0.072	2478	–1343
(cCt)(AM)_2_-1		2.584	1.526	179.8	0.092	2167	–1657
(cCt)(AM)_2_-2	C	2.727	1.763	161.8	0.030	3173	–657
	T	2.686	1.690	167.7	0.044	2921	–903

a The labels “C” and “T” have been indicated in Figure 4.

b Asymmetric stretching mode of the two O–H groups with intensity of 4171 km/mol. The calculated value of the symmetric stretching mode of the same bonds is 2892 cm^–1^ with an intensity of just 5.2 km/mol.

c Asymmetric stretching mode of the two O–H groups with intensity of 3785 km/mol. The calculated value of the symmetric stretching mode of the same bonds is 3023 cm^–1^ with a negligible intensity.

d Asymmetric stretching mode of the two O–H groups with intensity of 3831 km/mol. The calculated value of the symmetric stretching mode of the same bonds is 3023 cm^–1^ with an intensity of just 6.6 km/mol.

As can be seen, all five conformers of OA form strong O–H···N type HB with an average HB distance (angle) of 1.67 Å (168.4°) in the case of binary clusters and 1.64 Å (171.3°) in the case of ternary clusters. The average red shift of the OA O–H stretching frequency is 934 (1088) cm^–1^ in binary (ternary) clusters. Among the two conformations of (cTt)(AM) and also of (cCt)(AM), Conf. (1) with AM interacting with the single COOH moiety of OA shows stronger hydrogen bonding. For ternary clusters involving two AM molecules interacting with OA, we sought configurations that consistently allow interaction with both the OH and CO moieties of OA, originating from either the same or different COOH groups, which yielded two scenarios. The first one involves direct hydrogen bonding between the two AM molecules, while both interact with OA. In the second scenario, each AM molecule interacts separately with OA, remaining spatially distant from each other. Clusters formed under the second scenario exhibit higher energetic stability for all OA conformers, as can be verified from the relative electronic energy difference provided in Figure 4. Furthermore, in the second scenario, the vibrational degeneracy of the OH stretching mode persists as the symmetry of the system is maintained. Thus, in the cTc, tTt, and tCt conformers, the coupled asymmetric and symmetric stretchings of the two equivalent OH groups continue, both experiencing similar red-shifts. However, in the first scenario, where two AM molecules act jointly on one side of OA, the symmetry is altered and the degeneracy is lifted. In this case, only one of the OH groups directly forms HB by donating a proton to the nitrogen of AM, resulting in a substantial red-sift for that specific OH group.

In Table 3, we present the calculated binding electronic energy (Δ*E*), binding Gibbs free energy (Δ*G*) at 298.15 K along with the distortion energy (Δ*E*_D_) of the OA monomers and their corresponding binary and ternary clusters with AM. Table 3 additionally reports the population distributions within each cluster compositions, denoted as relative population fraction (RPF), the multiple-conformer cluster binding free energy, Δ*G*_MC_ in kcal/mol, for each composition and the equilibrium constants (*K*_eq_) at 298.15 K for each cluster.

**Table 3:** Calculated Values of Binding Electronic Energies (Δ*E*), Binding Free Energy (Δ*G*) Associated with Different (OA)(AM) and (OA)(AM)_2_ Clusters at 298.15 K, in kcal/mol, Along with their Relative Population Fraction (RPF), the Multi-Conformation Average Binding Free Energy (Δ*G*_*MC*_) and the Equilibrium Constants (*K*_*eq*_) of each Cluster Composition Obtained at M06–2*X*/6-311++G(3df,3pd) Level

	Δ*E*_B_	*E*_D_(OA)	*E*_D_(tot)	Δ*G*	RPF	Δ*G*_MC_	*K*_eq_
(OA)(AM)							
(cTc)(AM)	–11.89	4.32	4.39	–3.65	100.00	–3.65	4.8 × 10^2^
(cTt)(AM)-1	–12.76	3.16	3.22	–4.51	99.79	–4.51	2.1 × 10^3^
(cTt)(AM)-2	–9.73	1.84	1.88	–0.87	0.21		
(tTt)(AM)	–11.72	1.48	1.53	–3.39	100.00	–3.39	3.1 × 10^2^
(tCt)(AM)	–11.81	1.50	1.55	–2.71	100.00	–2.71	9.8 × 10^1^
(cCt)(AM)-1	–13.57	2.06	2.11	–5.03	99.68	–5.03	4.9 × 10^3^
(cCt)(AM)-2	–8.90	2.60	2.63	–1.63	0.32		
(OA)(AM)_2_							
(cTc)(AM)_2_-1	–21.33	5.91	6.05	–5.86	99.92	–5.86	2.0 × 10^4^
(cTc)(AM)_2_-2	–18.13	8.00	8.18	–1.66	0.08		
(cTt)(AM)_2_-1	–20.26	3.53	3.65	–3.68	89.15	–3.75	5.7 × 10^2^
(cTt)(AM)_2_-2	–19.37	4.65	4.84	–2.43	10.85		
(tTt)(AM)_2_-1	–22.42	2.19	2.31	–5.75	99.92	–5.75	1.7 × 10^4^
(tTt)(AM)_2_-2	–18.33	3.59	3.76	–1.52	0.08		
(tCt)(AM)_2_-1	–22.70	2.34	2.46	–5.43	99.88	–5.43	9.7 × 10^3^
(tCt)(AM)_2_-2	–18.74	3.56	3.73	–1.44	0.12		
(cCt)(AM)_2_-1	–21.21	5.12	5.20	–4.51	48.04	–4.95	4.3 × 10^3^
(cCt)(AM)_2_-2	–20.83	3.30	3.40	–4.56	51.96		

The structural data reported in Table 2 and the large negative electronic-binding energies reported in Table 3 demonstrate that all five conformers of OA can form stable hydrogen-bonded molecular clusters with AM under ambient conditions, and if the gas-phase molecular concentrations are sufficient, these clusters may further nucleate and grow in size. Among the binary clusters, the lowest binding energy is obtained for (cCt)(AM)-1 with Δ*E*_B_ = −13.57 kcal/mol, followed by (cTt)(AM)-1 with Δ*E*_B_ = −12.76 kcal/mol. Both cCt and cTt have the same Cs symmetry, and in both cases, AM interacts with one COOH group where the CO and OH are in cis configuration, Notably, neither cCt nor CTt is the lowest energy conformer of OA. In fact, cCt is the highest energy OA conformer with Δ*E*_R_*=* 5.78 kcal/mol relative to the most stable cTc conformer. The binary cluster formed by the lowest energy cTc conformer ranks third with Δ*E*_B_*= −*11.89 kcal/mol, closely followed by (tCt)(AM) and (tTt)(AM) with Δ*E*_B_ = −11.81 and −11.79 kcal/mol, respectively.

Concerning the total distortion energy, *E*_D_(*T*), of the binary clusters, it is found that (cTc)(AM) exhibits the highest value with *E*_D_(*T*) = 4.39 kcal/mol, which accounts for almost 37% of its binding energy, Δ*E*_B_. In contrast, the most stable (cCt)(AM)-1 binary conformer has *E*_D_(*T*) = −2.63 kcal/mol, constituting 19% of its Δ*E*_B_. The (tTt)(AM) binary cluster has the lowest distortion energy with *E*_D_(*T*) = 1.53 kcal/mol, followed very closely by (tCt)(AM) with *E*_D_(*T*) = 1.55 kcal/mol. In both clusters, *E*_D_(*T*) corresponds to 13% of Δ*E*_B_. Thus, the distortion energy of (cTc)(AM) is nearly three times that of (tCt)(AM), although their Δ*E*_B_ has almost the same magnitude. Furthermore, in all the binary clusters, the distortion energies of the individual OA monomers, *E*_D_(OA) contribute to 95–99% of *E*_D_(T), which implies that the OA monomers undergo the most structural modifications during clustering. So, the lowest energy OA conformer cTc experiences significantly higher structural strain compared to others, as its *E*_D_(*T*) is substantially higher than all other conformers. OA conformers with trans-COOH interacting with AM, suffer lesser distortion, benefiting from steric advantages over others.

Upon addition of thermal correction to electronic energy, all binary clusters exhibit negative values of binding free energy (Δ*G*), signifying the spontaneous formation of the cluster at ambient temperature and pressure, if monomer concentrations are sufficient at the specific location. The order of thermodynamic stability aligns with that of Δ*E*_B_, where the (cCt)(AM)-1 conformer exhibits the lowest Δ*G* value of −5.03 kcal/mol, followed by (cTt)(AM)-1 and (cTc)(AM) with higher values of −4.51 kcal/mol and −3.65 kcal/mol, respectively. The *K*_eq_ values were calculated by using the formula: where *R* = 8.314 J/(mol·K) is the universal gas constant and *T* = 298.15 K is the ambient temperature. The equilibrium constant of a chemical system is a measure of the proportion of products and reactants present in a given equilibrium state. In atmospheric particle cluster formation, this constant is closely linked to changes in Gibb’s free energy, with smaller free energy changes corresponding to larger equilibrium constants. This suggests that clusters are more likely to form and remain stable in the atmosphere. Hence, a higher equilibrium constant indicates a preference for cluster formation over dissociation.

For (cTc)(AM), (tTt)(AM), and (tCt)(AM), each having one conformation, Δ*G*_MC_*=* Δ*G*. On the other hand, (cTt)(AM) and (cCt)(AM) compositions possess two conformations each, but the Gibbs free energy difference between the two conformations exceeds 3 kcal/mol in both cases. As a result, in these two cases, the value of Δ*G*_MC_*=* Δ*G* of the more stable conformation This substantial free energy difference significantly influences the RPF of the clusters. The RPF of (cTc)(AM), (tTt)(AM), and (tCt)(AM) is 100% as they have one conformation each. However, in the case of (cTt)(AM) and (cCt)(AM), where the difference between the Δ*G* values of conformation-1 and conformation-2 exceeds 3 kcal/mol, conformation-1 is the dominant fraction retaining over 99% of the total population. Furthermore, as can be observed from Table 3, the (cCt)(AM) binary cluster has the highest equilibrium constant (*K*_eq_ = 4.9 × 10^3^), followed by (cTt)(AM) with *K*_eq_ = 2.1 × 10^3^, while (tCt)(AM) has the lowest value with *K*_eq_ = 9.8 × 10^1^ which implies that, under ambient conditions, the population of (cCt)(AM) cluster should be approximately 2.3 times greater than that of (cTt)(AM), and about 100 times greater than that of (tCt)(AM).

In the case of ternary clusters of OA with AM, a total of 10 clusters is considered, with each cluster composition having two conformations. Similar to the previous case, all clusters exhibit large negative values of Δ*E*_B_. However, they have a different profile this time, as the cCt and cTt conformers with Cs symmetry no longer constitute the most stable clusters, in terms of binding energy. Instead, the ternary cluster with the lowest Δ*E*_B_ is (tCt)(AM)_2_-1 with Δ*E*_B_ = *–*22.70 kcal/mol, closely followed by (tTt)(AM)_2_-1 with Δ*E*_B_ = −22.42 kcal/mol. In both of these clusters, the two AM monomers interact separately with the COOH moieties of OA, with no direct hydrogen bonding between them. The lower energy conformation, i.e., conformation-1 of the ternary clusters formed by cTc (the lowest energy OA conformer) and cCT (the highest energy OA conformer) have practically the same Δ*E*_B_ (around −21 kcal/mol), but the corresponding higher energy conformations, i.e., conformation-2 differ by almost 3 kcal/mol, with (cCt)(AM)_2_-2 having the lower Δ*E*_B_.

The total distortion energies, *E*_D_(*T*), of the two conformations of (cTc)(AM)_2_ generally have higher magnitudes compared to others, while the ternary clusters of tTt and tCt, both with C_2v_ symmetry, exhibit the lowest *E*_D_(*T*), as can be verified from the table. Similar to the binary clusters, distortion of the OA monomer alone in each ternary cluster contributes to 95–98% of *E*_D_(T). Regarding the Gibb’s free energy variation, all ternary clusters show negative values, with those of cTc, tTt and tCt, in particular, having the Δ*G*’s closely similar in magnitudes. Specifically, (cTc)(AM)_2_-1, (tTt)(AM)_2_-1, and (tCt)(AM)_2_-1 have Δ*G* in the range of −5.43 to −5.86 kcal/mol. On the other hand, the higher energy conformations, (cTc)(AM)_2_-2, (tTt)(AM)_2_-2, and (tCt)(AM)_2_-2 exhibit Δ*G*’s varying between −1.44 and −1.66 kcal/mol. Since the difference of Δ*G* between conformation-1 and conformation-2 of these cluster compositions exceeds 3 kcal/mol, it strongly impacts the RPF, rendering the population of conformation-2 negligible. However, in the case of (cCt)(AM)_2_, where the Δ*G* values of the two conformations differ only by 0.05 kcal/mol with conformation-2 having the lower value, the RPF reflects a more balanced distribution, with conformation-1 occupying approximately 48% and conformation-2 around 52%. Regarding the equilibrium constants of the ternary clusters, (cTc)(AM)_2_ exhibits the highest value (*K*_eq_ = 2.0 × 10^4^), closely followed by (tTt)(AM)_2_ with *K*_eq_ = 1.7 × 10^4^, suggesting that both should have similar relative populations under ambient conditions. Further considering the *K*_eq_ values of the other ternary clusters, it is evident that the population of (cTc)(AM)_2_ should be nearly double that of (tCt)(AM)_2_ and about five times greater than that of (cCt)(AM)_2_.

The previous discussion of the binding free energy of the clusters assumed that monomers interact simultaneously to form clusters. However, clusters can also form through successive interactions, where a preformed molecular cluster interacts with another free molecule to create a larger cluster. In our case, a ternary (OA)(AM)_2_ cluster may form from the interaction of a binary (OA)(AM) cluster with an AM monomer. Table 4 presents the different possible pathways for forming (OA)(AM)_2_ through successive cluster formation, which is relevant to the growth of the cluster size. Successive binding free energies (Δ*G*_s_) are calculated for all five ternary (OA)(AM)_2_ clusters, corresponding to the five OA conformers, assuming that any conformation of a ternary cluster composition can be formed from the hydrogen bonded interaction of one AM monomer with any of the corresponding binary composition.

**Table 4:** Successive Binding Free Energies (Δ*G*_S_) for the Formation of Various (OA)(AM)_2_ Ternary Clusters, Derived from the Addition of an AM Monomer to Pre-Existing (OA)(AM) Binary Clusters

Final channel (ternary cluster)	Initial channel (binary cluster + AM)	Δ*G*_S_ (kcal/mol)
(cTc)(AM)_2_-1	(cTc)(AM) + AM	–2.21
(cTc)(AM)_2_-2		1.99
(cTt)(AM)_2_-1	(cTt)(AM)-1 + AM	–2.81
	(cTt)(AM)-2 + AM	0.83
(cTt)(AM)_2_-2	(cTt)(AM)-1 + AM	–1.56
	(cTt)(AM)-2 + AM	2.08
(tTt)(AM)_2_-1	(tTt)(AM) + AM	–2.36
(tTt)(AM)_2_-2		1.87
(tCt)(AM)_2_-1	(tCt)(AM) + AM	–2.72
(tCt)(AM)_2_-2		1.27
(cCt)(AM)_2_-1	(cCt)(AM)-1 + AM	0.52
	(cCt)(AM)-2 + AM	–2.88
(cCt)(AM)_2_-2	(cCt)(AM)-1 + AM	0.47
	(cCt)(AM)-2 + AM	–2.93

In the case of simultaneous cluster formation, as shown in Table 3, all ten ternary structures show negative Δ*G* values, with conformation-1 consistently having a significantly lower Δ*G* than conformation–-2, except in (cCt)(AM)_2_. However, in successive cluster formation, not all binding free energies (Δ*G*_S_) are negative, indicating some selectivity or preference. For binary compositions with a single conformation—(cTc)(AM), (tTt)(AM), and (tCt)(AM)—only for the formation of (cTc)(AM)_2_-1, (tTt)(AM)_2_-1, and (tCt)(AM)_2_-1 shows negative Δ*G*_S_ values. These are the ternary conformations with a lower Δ*G* of simultaneous cluster formation within their respective compositions. On the other hand, for binary compositions with two conformations, only the one with lower Δ*G* is capable of forming a ternary cluster with negative Δ*G*_S_. For example, theoretically, both (cCt)(AM)_2_-1 and (cCt)(AM)_2_-2 could be formed from either (cCt)(AM)-1 or (cCt)(AM)-2 via successive cluster formation. However, since Δ*G*[(cCt)(AM)-2] < Δ*G*[(cCt)(AM)-1], the thermodynamically favorable cluster formation pathways are (cCt)(AM)-2 + AM → (cCt)(AM)_2_-1 with Δ*G*_s_ = −2.88 kcal/mol and (cCt)(AM)-2 + AM → (cCt)(AM)_2_-2 with Δ*G*_s_ = −2.93 kcal/mol. Thus, (cCt)(AM)-2 can spontaneously grow in size through successive cluster formation, while (cCt)(AM)-1 cannot. Similarly, (cTt)(AM)-1 can grow through successive cluster formation due to its lower Δ*G* value, while (cTt)(AM)-2 cannot.

### 3.3 Clusters of Oxalic Acid and Sulfuric Acid, (OA)(SA)_*n*_ (*n* = 1,2)

**Figure 5:**
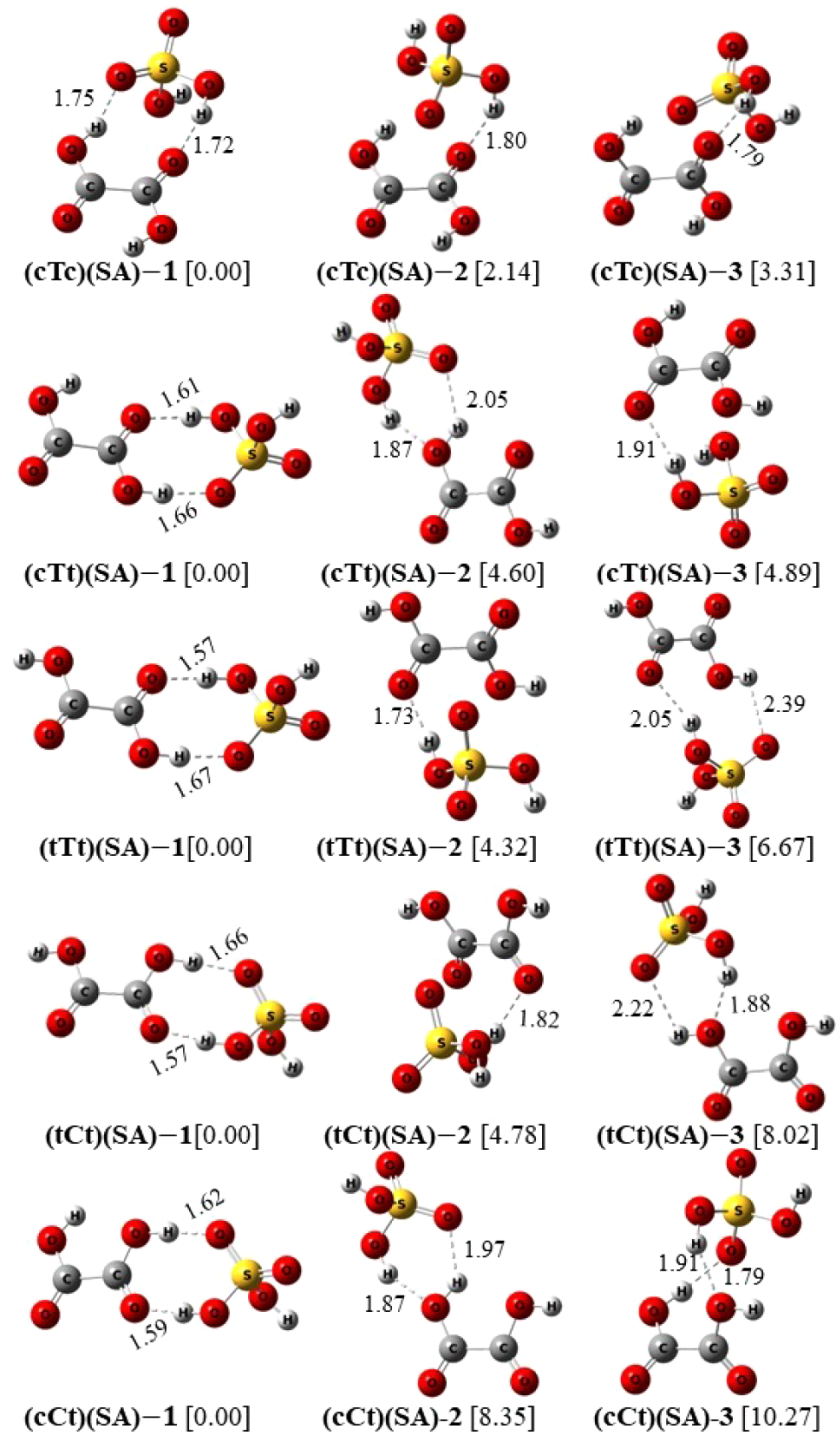
Equilibrium geometries of stable (OA)(SA) cluster compositions. optimized at the MO6–2*X*/6-311++G(3df,3pd) level. The dashed lines represent the intermolecular hydrogen bonds with respective bond lengths given in angstrom. The numbers in square brackets represent the relative energy differences of the conformations in each cluster composition, in kcal/mol.

**Figure 6:**
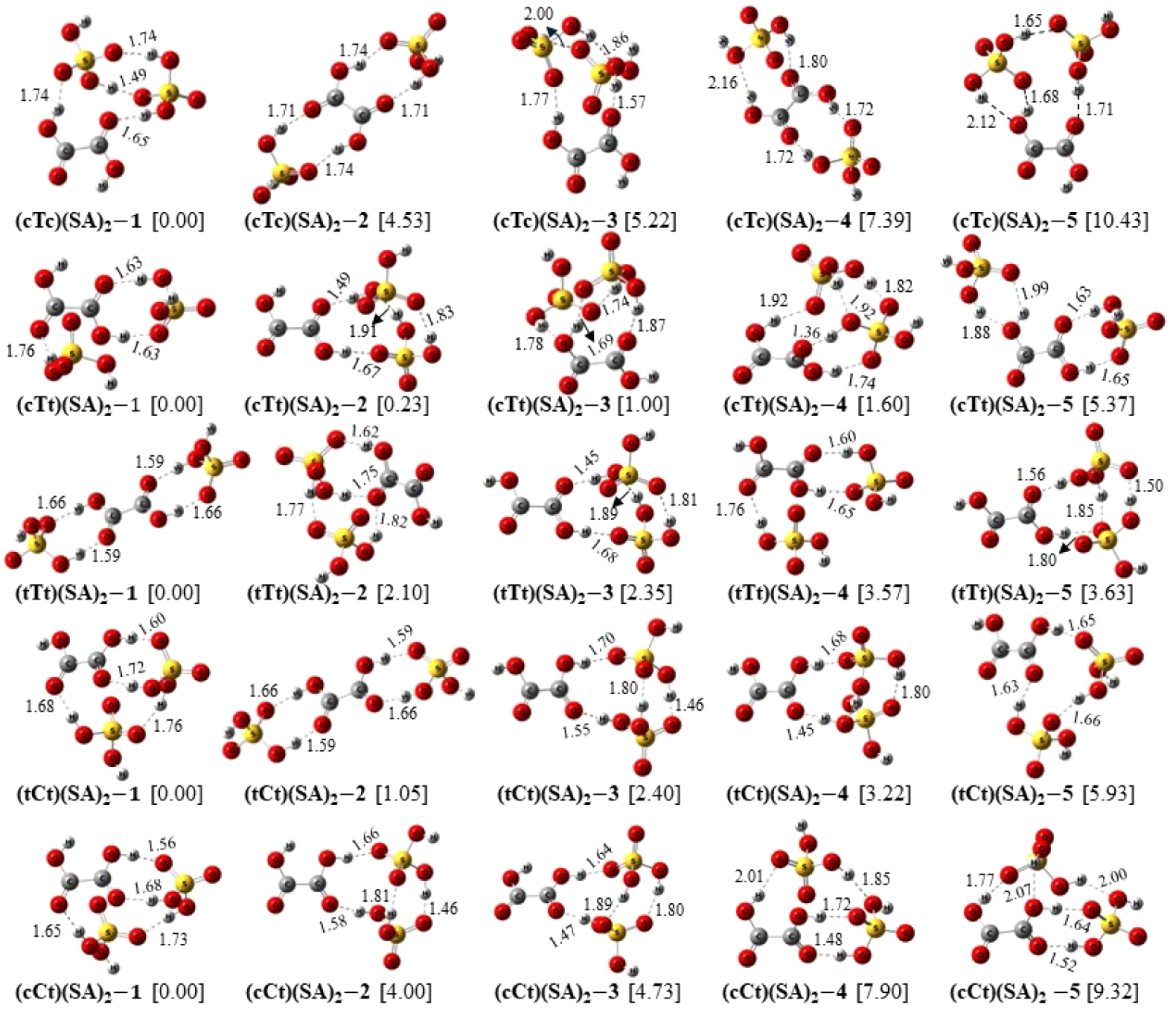
Equilibrium geometries of the stable (OA)(SA)_2_ cluster compositions. optimized at the MO6–2*X*/6-311++G(3df,3pd) level. The dashed lines represent the intermolecular hydrogen bonds with respective bond lengths given in angstrom. The numbers in square brackets represent the relative energy differences of the conformations in each cluster composition, in kcal/mol.

In this section, we analyze the structural and thermochemical properties of the binary and ternary clusters formed by each of the five conformers of OA (cTc, cTt, tTt, tCt, cCt) with SA at ambient condition. The configurational space of the OA–SA system is larger than that of OA–AM and we have larger numbers of cluster conformations for each OA conformation in this case. In order to be concise, we have selected three (five) lowest energy conformations of each binary (ternary) cluster composition in the present work. Figure 5 exhibits the optimized geometries of the 15 binary (OA)(SA) clusters, with each OA conformer having three conformations. Figure 6 displays the optimized structures of 25 ternary (OA)(SA)_2_ cluster conformations, with five for each OA conformer. Each row in the figures corresponds to the conformations of a particular composition, labeled according to the respective OA conformer nomenclature. The relative energy differences of the conformations, in kcal/mol, are indicated in square bracket. The intermolecular HBs are indicated by black dashed lines, with respective calculated bond lengths in angstroms. The M06–2*X*/6-311++G(3df,3pd) optimized structure of (cTc)(SA)-1, which is the global minimum of the (cTc)(SA) conformers has an excellent agreement with binary (OA)(SA) global minimum obtained by PW91PW91/6-311++G(3df,3pd) model.^8^

As can be seen from Figure 5, the HB lengths of the HBs vary between 1.57 and 2.39 Å, with more than 80% of them remaining below 2.00 Å. Unlike the binary (OA)(AM) clusters, the HBs in (OA)(SA) clusters, with OA being the proton donor, are longer than those where OA acts as proton acceptor. Moreover, the differences between the bond lengths of these two types of HBs are also considerably smaller than those in (OA)(AM) systems. For example, in (cTc)(AM), we observe *R*_O–H···N_ = 1.62 Å (OA as proton donor) and *R*_N–H···O_ = 2. 25 Å (OA as proton acceptor), while in (cTc)(SA), considering its lowest energy conformation, *R*_O–H···O_ = 1.75 Å (OA as proton donor) and *R*_O–H···O_ = 1.72 Å (OA as proton acceptor), with *R* denoting the HB length. Similar trends are observed in other clusters as well. The HB bond angles are also similar in the case of the two – H···O HBs in the latter case. Thus, in the case of (OA)(SA) clusters, both OA and SA contribute equivalently acting as simultaneous proton donor and acceptor. The Cartesian coordinates of all the optimized geometries of OA)(SA) and (OA)(SA)_2_ clusters are given in Table S3a,b, respectively. In Table S4, we report the relevant structural and spectroscopic parameters for the HBs present in (OA)(SA) binary clusters.

As illustrated in Figure 6, the ternary (OA)(SA)_2_ clusters, in general, are stabilized by the formation of three-five intermolecular HBs. Depending on the positions of the two SA molecules around OA, the later may have direct participation in the formation of two to four HBs. The O–H···O HB lengths range between 1.35 and 2.16 Å, with 93% of them remaining below 2.00 Å and almost 70% below 1.80 Å. The average HB angles of the ternary cluster compositions, except (cTc)(SA)_2_, are larger than those of corresponding binary clusters. For example, the average HB angle of the binary (tCt)(SA) conformers is 154.8°, while in (tCt)(SA)_2_, it is 174.2°. In (cTc)(SA) and (cTc)(SA)_2_ the average bond angle remains around 161°. In Table S5, we report the relevant structural and spectroscopic parameters for the HBs present in (OA)(SA)_2_ clusters.

The proton-donor O–H groups of both OA and SA suffer a strong red-shift upon cluster formation. If we consider the lowest energy conformation of each binary (OA)(SA) cluster compositions, the average red-shift suffered by OH of OA is 504 cm^–1^, with cTc being the only OA conformer having a red-shift below this average. On the other hand, the average red-shift experienced by the OH of SA in these same systems is 712 cm^–1^. Thus, although cTc is the lowest energy OA monomer, its binary clusters with SA demonstrate weaker HB strength compared to others, and in all clusters, SA appears as a stronger proton-donor. However, in ternary (OA)(SA)_2_ clusters, the average red-shift OH of OA (∼ 630 cm^–1^) is almost same as that of OH of SA (∼ 620 cm^–1^) showing that OA participates more effectively in HB formation in ternary clusters.

Regarding the energetics, all clusters of OA with SA display large negative Δ*E*_B_ values, as can be verified from the data reported in Tables 5 and 6. Among the conformations within each cluster composition, there is considerable variation in Δ*E*_B_. Considering the average binding energy, ⟨Δ*E*_B_⟩ of each cluster composition in Table 5, the binary (OA)(SA) clusters can be arranged in the following order of increasing ⟨Δ*E*_*B*_⟩: (tTt)(SA) [−13.62] <; (tCt)(SA) [−13.34] <; (cTt)(SA) [−12.94] <; (cCt)(SA) [−11.82] <; (cTc)(SA) [−11.05], where the numbers in the square brackets represent the values of ⟨Δ*E_B_⟩* in kcal/mol. In case of ternary (OA)(SA)_2_ clusters, this order is different, and it is as follows: (cCt)(SA)_2_ [−35.51] <; (tCt)(SA)_2_ [−33.24] <; (tTt)(SA)_2_ [−31.92] <; (cTt)(SA)_2_ [−28.74] <; (cTc)(SA)_2_ [−27.33] from Table 6. However, when considering the lowest energy conformer of each cluster composition, among the binary clusters, (cCt)(SA)-1 exhibits the least binding energy with Δ*E*_B_ = −18.03 kcal/mol, while (cTc)(SA)-1 shows the highest value with Δ*E*_B_ = −13.69 kcal/mol. Notably, among the five OA conformers, cTc has the lowest electronic energy, while cCt has the highest. Thus, like the case of (OA)(AM) clusters, the highest energy OA conformer also forms a binary cluster with SA that has the lowest binding energy among all cluster compositions. The binding energy of (cCt)(SA)-1 is closely followed by (tCt)(SA)-1 and (tTt)(SA)-1 with Δ*E*_*B*_ values of −17.61 kcal/mol and −17.28 kcal/mol, respectively. In case of the ternary clusters also, the highest energy OA conformer forms the cluster (cCt)(SA)_2_-1, that possesses the least binding energy with Δ*E*_B_ = −37.70 kcal/mol.

**Table 5:** Calculated Values of Binding Electronic Energies (Δ*E*), Binding Free Energy (Δ*G*) Associated with Different (OA)(SA) Clusters at 298.15 K, in kcal/mol, Along with their Relative Population Fraction (RPF), the Multi-Conformation Average Binding Free Energy (Δ*G*_MC_) and the Equilibrium Constants (*K*_eq_) of each Cluster Composition Obtained at M06-2*X*/6-311++G(3df,3pd) Level

	Δ*E*_*B*_	*E*_D_(OA)	*E*_D_(tot)	Δ*G*	RPF	Δ*G*_MC_	*K*_*eq*_
(cTc)(SA)-1	–13.69	1.76	2.70	–3.39	99.10		
(cTc)(SA)-2	–11.54	1.09	1.96	–0.58	0.88	–3.39	3.1 × 10^2^
(cTc)(SA)-3	–7.91	0.34	0.62	1.67	0.02		
(cTt)(SA)-1	–16.10	1.39	3.42	–5.30	99.70		
(cTt)(SA)-2	–11.50	1.62	2.27	–1.84	0.29	–5.30	7.8 × 10^3^
(cTt)(SA)-3	–11.21	1.55	3.99	0.24	0.01		
(tTt)(SA)-1	–17.28	1.48	3.94	–6.69	99.99		
(tTt)(SA)-2	–12.97	0.66	1.61	–1.30	0.01	–6.69	8.1 × 10^4^
(tTt)(SA)-3	–10.61	1.05	1.98	–0.26	0.00		
(tCt)(SA)-1	–17.61	1.53	4.13	–6.26	99.99		
(tCt)(SA)-2	–12.83	0.49	1.17	–0.41	0.01	–6.26	3.9 × 10^4^
(tCt)(SA)-3	–9.58	0.61	0.95	2.41	0.00		
(cCt)(SA)-1	–18.03	1.74	4.40	–7.04	100.00		
(cCt)(SA)-2	–9.68	0.35	0.81	0.13	0.00	–7.04	1.5 × 10^5^
(cCt)(SA)-3	–7.76	4.25	5.07	2.95	0.00		

**Table 6:** Calculated Values of Binding Electronic Energies (Δ*E*), Binding Free Energy (Δ*G*) Associated with Different (OA)(SA)_2_ Clusters at 298.15 K, in kcal/mol, Along with their Relative Population Fraction (RPF), the Multi-Conformation Average Binding Free Energy (Δ*G*_*MC*_) and the Equilibrium Constants (*K*_*eq*_) of each Cluster Composition Obtained at M06-2*X*/6-311++G(3df,3pd) Level

	Δ*E*_*B*_	*E*_D_(OA)	*E*_D_(*T*)	Δ*G*	RPF	Δ*G*_MC_	*K*_*eq*_
(cTc)(SA)_2_-1	–32.84	1.92	12.57	–9.65	96.61		
(cTc)(SA)_2_-2	–28.32	2.95	5.02	–7.67	3.38		
(cTc)(SA)_2_-3	–27.62	2.19	7.23	–4.20	0.01	–9.67	1.3 × 10^7^
(cTc)(SA)_2_-4	–25.46	2.76	4.79	–2.92	0.00		
(cTc)(SA)_2_-5	–22.42	2.00	7.45	–0.32	0.00		
(cTt)(SA)_2_-1	–30.38	1.98	5.12	–7.01	60.15		
(cTt)(SA)_2_-2	–30.15	1.91	8.58	–6.66	33.10		
(cTt)(SA)_2_-3	–28.78	1.71	6.41	–5.26	3.15	–7.31	2.3 × 10^5^
(cTt)(SA)_2_-4	–29.38	10.39	23.55	–5.28	3.23		
(cTt)(SA)_2_-5	–25.01	1.64	4.01	–4.00	0.37		
(tTt)(SA)_2_-1	–34.25	2.96	7.56	–11.82	99.43		
(tTt)(SA)_2_-2	–32.15	3.28	11.42	–7.70	0.10		
(tTt)(SA)_2_-3	–31.90	2.17	9.81	–8.56	0.40	–11.83	4.8 × 10^8^
(tTt)(SA)_2_-4	–30.68	1.89	5.09	–7.11	0.03		
(tTt)(SA)_2_-5	–30.63	1.25	10.04	–7.19	0.04		
(tCt)(SA)_2_-1	–35.76	3.29	13.57	–10.08	6.53		
(tCt)(SA)_2_-2	–34.71	2.90	7.59	–11.65	92.27		
(tCt)(SA)_2_-3	–33.36	1.63	17.43	–8.91	0.91	–11.70	3.9 × 10^8^
(tCt)(SA)_2_-4	–32.54	2.26	10.05	–8.25	0.30		
(tCt)(SA)_2_-5	–29.83	1.71	7.28	–5.22	0.00		
(cCt)(SA)_2_-1	–37.70	4.36	13.19	–13.31	99.41		
(cCt)(SA)_2_-2	–33.70	1.81	17.50	–10.16	0.49		
(cCt)(SA)_2_-3	–32.98	2.40	9.77	–9.24	0.10	–13.31	5.9 × 10^9^
(cCt)(SA)_2_-4	–29.80	2.92	8.67	–5.83	0.00		
(cCt)(SA)_2_-5	–28.38	5.78	11.18	–4.57	0.00		

Considering the thermal correction to electronic energy, we observe that not all binary cluster conformers show thermodynamic stability at ambient temperature. In the case of the three conformers of (cCt)(SA) composition, for example, only (cCt)(SA)-1 has negative Δ*G*, and it is also the one with lowest binding free energy among all the binary clusters considered here, with Δ*G =* −7.04 kcal/mol. Only binary composition whose all conformations show negative Δ*G* values at room temperature is (tTt)(SA), with (tTt)(SA)-1 having the binding free energy very close to (cCt)(SA)-1 with Δ*G =* −6.69 kcal/mol. The binary cluster of cTc, the lowest energy OA monomer, with SA shows the highest Δ*G* value. The ternary clusters of OA with SA, however, show a different nature where all the five conformations of each cluster composition show negative Δ*G* valuess of varying magnitudes at 298.15K. The lowest energy conformation of each cluster composition has the lowest Δ*G* of the respective group except (tCt)(SA)_2_ where second lowest conformer (tCt)(SA)_2_-2 shows lowest value with Δ*G* = −11.65 kcal/mol, followed by (tCt)(SA)_2_-1 with Δ*G* = −10.08 kcal/mol. Considering the Δ*G*_MC_ values, the ternary clusters can be arranged in the order of increasing thermodynamical stability as follows: (cTt)(SA)_2_ <; (cTc)(SA)_2_ <; (tCt)(SA)_2_ <; (tTt)(SA)_2_ <; (cCt)(SA)_2_. Thus, in both the binary and ternary clusters of OA with SA, cCt, the highest energy OA conformer, forms the most thermodynamically stable interaction with the lowest Δ*G* values. Overall, the Δ*G* values of the (OA)(SA)_2_ clusters are much lower than those of the binary (OA)(SA) clusters, indicating higher stability for the ternary clusters.

Regarding the relative population fraction (RPF) of the binary (OA)(SA) clusters, a similar trend to that observed in (OA)(AM) clusters is observed, with the lowest energy conformer of each cluster dominating the population with an RPF of nearly 100%. In the case of (OA)(SA) trimers, a similar trend is observed, with the lowest Δ*G* conformer of each cluster composition having the dominant RPF. This dominance is over 90% in all cases except for (cTt)(SA)_2_, where due to the small difference in Δ*G* values between (cTt)(SA)_2_-1 and (cTt)(SA)_2_-2, the RPFs of these two conformers are 60.15% and 33.10%, respectively.

Both in the binary and ternary clusters of OA with SA, the conformers cCt, tTt, and tCt of OA form the three most stable clusters, as indicated by their low Δ*G* values, which correspond to the highest equilibrium constants. Among the binary clusters, (cCt)(SA) shows the highest equilibrium constant (*K*_eq_ = 1.5 × 10^5^), followed by (tTt)(SA) and (tCt)(SA) with *K*_eq_ values of 8.1 × 10^4^ and 3.9 × 10^4^, respectively. Therefore, under ambient conditions, the (cCt)(SA) population should be nearly double that of (tTt)(SA) and about three times greater than that of (tCt)(SA). For the ternary clusters, [(cCt)(SA)_2_ shows the highest equilibrium constant (*K*_eq_ = 5.9 × 10^9^) which is 13 and 15 times higher than those (tTt)(AM)_2_ and (tCt)(AM)_2_, respectively.

Similarly to the (OA)(AM)_2_ clusters, an analysis of successive cluster formation was conducted for the (OA)(SA)_2_ clusters under the same theoretical assumption that any conformation of a ternary cluster composition can form through the hydrogen-bonded interaction between an SA monomer and any corresponding binary cluster. The calculated values of successive binding free energies (Δ*G*_S_) for all conformations of the five ternary (OA)(SA)_2_ cluster compositions, corresponding to the five OA conformers, are presented in Table S5. As seen from the table, unlike the case of (OA)(AM)_2_ clusters, the selectivity is much less pronounced in the successive formation of (OA)(SA)_2_ clusters, with the majority of successive interactions yielding negative Δ*G*_S_ values. This suggests that clusters of SA with any OA conformer can grow in size more readily than the corresponding AM clusters.

### 3.4 Atmospheric Relevance of the Binding Free Energies

Determining the concentrations of various binary and ternary clusters of OA with AM and SA under realistic atmospheric conditions is of interest, regarding the atmospheric relevance of these systems. These concentrations can serve as potential indicators of their presence in the atmosphere. The equilibrium constants (*K*_eq_) for the formation of these clusters from simultaneous agglomeration of the respective monomers, derived from their standard multiple-component binding free energies (Δ*G* at 298.15 K and 1 atm) and presented in Tables 3, 5, and 6, can be utilized for this analysis.

As has been discussed previously in the literature,^5−8^*K*_eq_ can also be defined for a cluster formation reaction like OA + *nX*→(OA)(*X*)*_n_* as
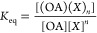


In the present case, *X* = AM or SA and *n =* 1,2. [OA], [*X*], and [(OA)(X)*_n_*] are the vapor pressures of OA, *X,* and their cluster (OA)(*X*)_*n*_, respectively. With this we can determine the percentage population fraction (%PF) of the OA clusters with respect to the OA monomer as,
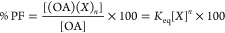


Baesd on the atmospherically relevant gas-phase concentrations of OA, AM, and SA, which are 5.0 × 10^11^, 2.5 × 10^10^, and 5.0 × 10^7^ molecules/cm^3^, respectively, as reported in the literature,^5−8^ we calculate the determine the %PF and the concentration of all the binary and ternary OA-clusters at the standard atmospheric condition of 298.15K and 1 atm, as reported in Table 7.

**Table 7:** Calculated Values of the Percentage Population Fraction (%PF) and Estimated Concentrations [C], in molecules/cm^3^, for Different Binary and Ternary (OA)(AM) and (OA)(SA) Cluster Compositions at 298.15K and 1 atm

AM-containing cluster	%PF	[C]	SA-containing cluster	%PF	[C]
(cTc)(AM)	4.77 × 10^–5^	2.39 × 10^5^	(cTc)(SA)	6.24 × 10^–8^	3.12 × 10^2^
(cTt)(AM)	2.04 × 10^–4^	1.02 × 10^6^	(cTt)(SA)	1.57 × 10^–6^	7.87 × 10^3^
(tTt)(AM)	3.08 × 10^–5^	1.54 × 10^5^	(tTt)(SA)	1.65 × 10^–5^	8.25 × 10^4^
(tCt)(AM)	9.75 × 10^–6^	4.87 × 10^4^	(tCt)(SA)	7.98 × 10^–6^	3.99 × 10^4^
(cCt)(AM)	4.92 × 10^–4^	2.46 × 10^6^	(cCt)(SA)	2.98 × 10^–5^	1.49 × 10^5^
(cTc)(AM)_2_	2.00 × 10^–12^	9.99 × 10^–3^	(cTc)(SA)_2_	5.15 × 10^–15^	2.58 × 10^–5^
(cTt)(AM)_2_	5.85 × 10^–14^	2.83 × 10^–4^	(cTt)(SA)_2_	9.55 × 10^–17^	4.77 × 10^–7^
(tTt)(AM)_2_	1.66 × 10^–12^	8.30 × 10^–3^	(tTt)(SA)_2_	1.98 × 10^–13^	9.91 × 10^–4^
(tCt)(AM)_2_	9.66 × 10^–13^	4.83 × 10^–3^	(tCt)(SA)_2_	1.59 × 10^–13^	7.96 × 10^–4^
(cCt)(AM)_2_	4.29 × 10^–13^	2.15 × 10^–3^	(cCt)(SA)_2_	2.42 × 10^–12^	1.21 × 10^–2^

As can be seen from the table, the binary clusters show atmospherically relevant concentrations, and it varies in the range of 10^4^ – 10^6^ molecules/cm^3^ for (OA)(AM) and 10^2^ – 10^5^ molecules/cm^3^ for (OA)(SA) compositions. Thus, some of the binary cluster concentrations are comparable to gas-phase SA concentrations. The estimated concentration of 2.39 × 10^5^ molecules/cm^3^ for (cTc)(AM) is also comparable with 8.02 × 10^5^ molecules/cm^3^ obtained for the same system previously by PW91PW91/6-311++G(3df,3pd) level of theory.^7^ Calculated concentrations of the ternary clusters are considerably smaller, with the (cCt)(SA)_2_ composition showing the maximum value.

Although the binding free energies calculated at standard atmospheric conditions are useful to assess the thermodynamical stability of the molecular clusters from quantum thermochemistry point of view, they may not be sufficient to evaluate their atmospheric relevance as no atmospherically relevant molecules actually have a partial pressure of 1 atm.^84^ Correction of Δ*G* by considering the effect of partial pressure of the reactant species may provide more realistic insight regarding the atmospheric relevance of these interactions which can be accomplished by the following general expression:^84^
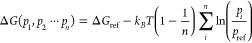


Here, *n* is the number of different monomers in the cluster, and *p*_*i*_ is the partial pressure of monomer *i*. In the present case, *n* = 2 as we consider the clusters of OA conformers either with AM or with SA, *p*_ref_ = 1 atm and Δ*G*_ref_ = Δ*G*_MC_, calculated at standard temperature and pressure and reported in Tables 3, 5, and 6. The above expression then reduces to
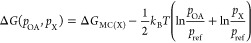


Where, *X* represents AM or SA monomer, *p*_OA_ is the partial pressure of OA, and *p*_X_ is the partial pressure of either AM or SA. Δ*G*_MC(*X*)_ denotes the Δ*G*_MC_ values for clusters containing either AM or SA, depending on whether X corresponds to AM or SA. For the gas-phase concentrations of OA, AM, and SA, mentioned above, the second term of the above equation evaluates to −11.26 and −13.01 kcal/mol, when *X* is AM and SA, respectively. Thus, for Δ*G*(*p*_OA_, *p*_X_) to be negative at 298.15K, indicating spontaneous cluster formation under atmospherically relevant conditions, Δ*G*_MC_ values of the (OA)(AM)_*n*_ and (OA)(SA)_*n*_ clusters must be lower than −11.26 and −13.08 kcal/mol, respectively. Thus, from the Δ*G*_MC_ values for simultaneous cluster formation reported in Tables 3, 5,and 6, we observe that (cCt)(SA)_2_, with Δ*G*_MC_ = −13.31 kcal/mol, is the only cluster composition which is thermodynamically stable in realistic atmospheric conditions at 298.15K. This is the same composition that showed highest concentration among the ternary clusters.

### 3.5 Interaction with Solar Radiation

The molecules present in the atmosphere interact with solar radiation. In fact, the elastic and inelastic scattering of solar radiation by atmospheric particles plays a significant role in understanding various phenomena related to visibility and radiative forces in the atmosphere. Elastic scattering of light, also known as Rayleigh scattering, stands out as the predominant optical phenomenon for small atmospheric molecular clusters, playing a vital role in various atmospheric processes. The intensity of Rayleigh scattering, often termed Rayleigh activity, depends on the dipole polarizability of the molecular system and its anisotropy. Polarizability (α) is a measure of how easily the electron cloud of a molecule can be distorted by an external electric field, that results in the creation of an induced dipole moment in the molecule. Anisotropy of polarizability refers to the directional dependence of the polarizability. Together, these characteristics govern the extent of interaction between incident radiation and the molecules, thereby influencing the scattering intensity. This fundamental interplay sheds light on the intricate dynamics of light-matter interactions within the atmosphere, providing insights into their complex behavior and processes. The formation of hydrogen-bonded molecular clusters can significantly affect the Rayleigh scattering intensity compared to their respective monomers due to variations in polarizability and anisotropy besides different cooperative effects.

**Figure 7:**
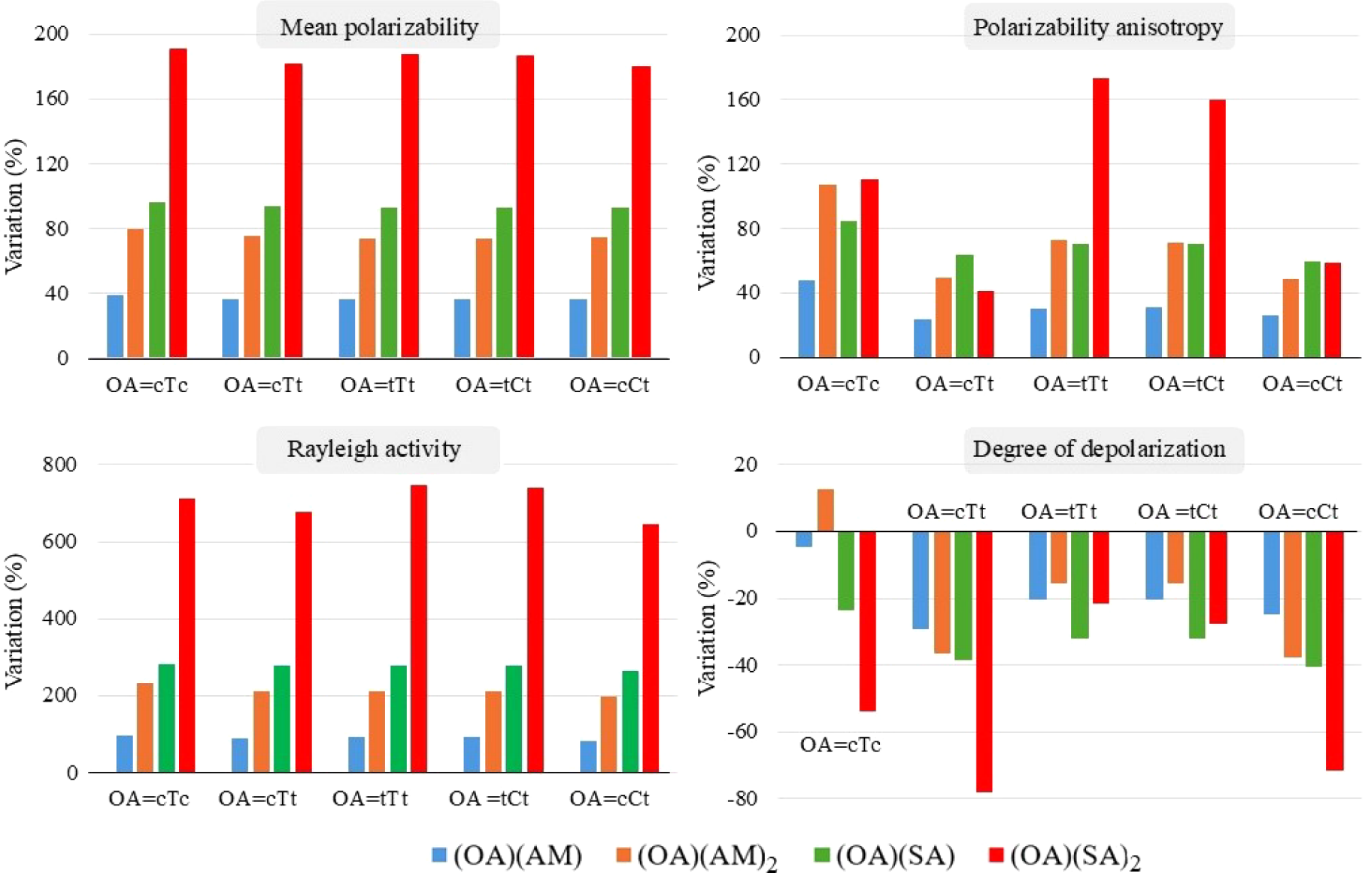
Percentage variation of mean dipole polarizability, anisotropy of the polarizability, Rayleigh activity, and degree of depolarization for natural light in all the clusters, relative to respective OA monomers.

In Figure 7, we present the percentage variation of mean dipole polarizability , anisotropy of the polarizability (Δα), Rayleigh Activity (), and degree of depolarization for natural light (σ) in all the clusters, relative to respective OA monomers. The values of these parameters for the monomers are reported in Table 1. Given that the clusters exhibit multiple conformations, we calculated the weighted average of each parameter (*x*) for each cluster composition by considering the Boltzmann factor of the respective members, exp(-Δ*G*_*k*_/*RT*) and using the formula
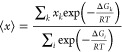
where *R* = 8.314 J/(mol·K) is the universal gas constant, *T* is the ambient temperature, and Δ*G*_*k*_ is the binding free energy of the *k*th member of a given cluster composition. These average values were then used to evaluate the percentage variations.

Since polarizability depends on molecular volume, it is expected that polarizability will increase upon cluster formation. This is confirmed by the figure, which shows an almost linear increase in mean polarizability as we progress from OA to (OA)(AM) or (OA)(SA) and then to (OA)(AM)_2_ or (OA)(SA)_2_ with OA representing any of the five OA monomers. Given that SA has a larger volume than AM, clusters of OA with SA show much higher increase in mean polarizability compared to clusters of AM with OA. For all OA monomers, polarizability increases by nearly 37% when they form binary clusters with AM and by almost 94% when forming binary clusters with SA. Similarly, in ternary clusters, the increase in mean polarizability is almost 75% for interaction with AM and 185% when interacting with SA. Considering the weighted average value of mean polarizability, , for each cluster composition individually, we find that the values are quite similar for five OA conformers. However, the anisotropy of polarizability does not exhibit the same regular and linear increase pattern as that of polarizability, although it increases upon cluster formation in all cases. Maximum increase of anisotropy, compared to the OA monomer, is observed for (tTt)(SA)_2_, followed very closely by (tCt)(SA)_2_. Conversely, the binary clusters formed between OA conformers and AM show less variation in Δα, consistently remaining below 50% in all cases. The average degree of depolarization (<*σ > ,*) decreases in all clusters, except (cTc)(AM)_2_, when compared to the respective OA monomers. Among the ternary OA–AM clusters, (cTc)(AM)_2_ possesses the highest polarizability with α = 67.24 au as well as the highest anisotropy with Δα = 42.23 au, while the average α and Δα for this ternary group are 66.51 and 31.76 au, respectively. Consequently, the < *σ >* value of (cTc)(AM)_2_ is high compared to others. The increase in the weighted average value of Rayleigh intensity (<  >) in the ternary (OA)(SA)_3_ clusters with respect to the OA monomer (exceeding 600%) is significantly higher than that of all other clusters. For the (OA)(AM)_2_ and (OA)(SA) cluster configurations, the increase of <  > ranges from 250% to 350% compared to the respective OA monomers, while in all (OA)(AM) binary clusters, the increase is limited to 100% or less.

**Figure 8:**
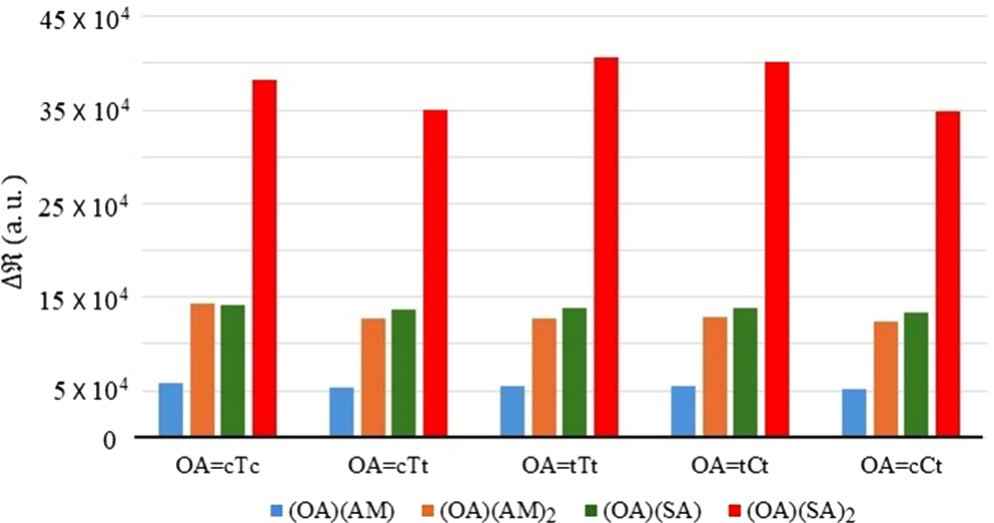
Excess Rayleigh scattering intensity (Δ) due to clustering of OA with AM and SA.

We can also analyze the increase in the Rayleigh scattering intensities due to clustering relative to all participating monomers using a supermolecular approach, where the excess Rayleigh intensity (Δ) will be determined by taking the difference in the scattering intensity between the cluster and the sum of the individual molecular intensities. Considering the weighted average value of Rayleigh intensity (<  >) of the clusters and the  of the corresponding monomers, we again observe an appreciable of Rayleigh intensity increase upon clustering in all cases which is illustrated in Figure 8.

In all the binary (OA)(AM) clusters, Δ values remain consistently close to an average of 5.4 × 10^4^ au, indicating that all five OA conformers interacting with an ammonia molecule via hydrogen bonding acquire similar molecular volumes, which leads to comparable polarizability and thus similar Rayleigh activities. A similar trend is observed in the ternary (OA)(AM)_2_ clusters, albeit with a higher average Δ of 13.0 × 10^4^ a.u., reflecting that the intermolecular interactions increase consistently across all the compositions of ternary (OA)(AM)_2_ clusters. The binary (OA)(SA) clusters also show a consistent increase in Rayleigh activity, with Δ values very similar to those of the (OA)(AM)_2_ clusters. In fact, (cTc)(AM)_2_ and (cTc)(SA) exhibit identical increases in Rayleigh activity upon cluster formation, with Δ = 14.2 × 10^4^ a.u., suggesting that sulfuric acid interacts more strongly with oxalic acid than ammonia does. Moreover, this value also represents the highest increase in Rayleigh activity within both the (OA)(AM)_2_ and (OA)(SA) families. The Δ values of ternary (OA)(SA)_2_ clusters are significantly higher than those of all other clusters, with an average value of 37.7 × 10^4^ a.u. Unlike the other cluster families, the Δ values of (OA)(SA)_2_ show appreciable composition dependence, with (cCt)(SA)_2_ having the lowest Δ (34.8 × 10^4^ a.u.) and (tTt)(SA)_2_ cluster the highest (40.5 × 10^4^ a.u.). This large increase in Δ (OA)(SA)_2_ compared to (OA)(SA) clusters indicates that the increase of sulfuric acid molecules leads to significantly stronger intermolecular interactions.

On a different perspective, comparison of <  > values among the four cluster families—(OA)(AM), (OA)(AM)_2_, (OA)(SA), and (OA)(SA)_2_—each having five members (compositions corresponding to the five OA conformers), either by composition or size, also reveals appreciable variations, as can be verified from the data reported in Table S6. For example, Considering the increase in cluster size, the average increase in  is 97% for (OA)(AM)_2_ ternary clusters and 114% for (OA)(SA)_2_ ternary clusters compared to their respective binary clusters—(OA)(AM) and (OA)(SA). Among the (OA)(AM) clusters, the highest increase (117%) is observed in (cTc)(AM)_2_, while the lowest (50%) occurs in (cCt)(AM)_2_. In case of (OA)(SA)_2_, all compositions show over 100% increase in  values going from binary to ternary cluster, with (tTt)(SA)_2_, showing the highest increase (124%) while (cTt)(SA)_2_ and (cCt)(SA)_2_, showing lowest increase, both with 105%.

When the compositions are compared, clusters of OA with SA consistently show higher Rayleigh intensity than the clusters of OA with AM due to their larger molecular volume. On average, the  values in binary (OA)(SA) and ternary (OA)(SA)_2_ clusters are higher than their corresponding (OA)(AM) and (OA)(AM)_2_ counterparts by 98% and 118%, respectively. However, there is a big difference in the individual behaviors of the members. However, significant variation is observed among the individual members. In the binary clusters, the largest difference is seen between (cTt)(SA) and (cTt)(AM), with the former’s  value being 99% higher, while the smallest variation of 96% is found between (cTc)(SA) and (cTc)(AM), indicating a consistent behavior with only a 3% difference between the maximum and minimum increases. On the other hand, for ternary clusters, the largest difference is observed between (cCt)(SA)_2_ and (cCt)(AM)_2_, with the former having 170% higher  value, while the smallest difference, 91%, is observed between (OAA)(SA)_2_ and (OAA)(AM)_2_. Other ternary clusters in this series— (cTt)(SA)_2_, (tTt)(AM)_2_, and (tCt)(AM)_2_—show increases of 111%, 110%, and 107%, respectively, with respect to their corresponding (OA)(AM)_2_ counterparts. These variations reflect considerable variation in molecular volume among ternary (OA)(SA) clusters

Finally, examining the four cluster families individually, we observe that the OA conformer cTc forms the clusters with the highest Rayleigh activity in all families except for (OA)(SA)_2_, It is (tTt)(SA)_2_ that has the highest  value in the (OA)(SA)_2_ family. Within the (OA)(AM) and (OA)(AM)_2_ families, the lowest  is observed in (cTt)(AM) and (cCt)(AM)_2_, respectively, and within the (OA)(SA) and (OA)(SA)_2_ families, (cCt)(SA) and (cTt)(SA)_2_ exhibit the lowest values. The difference between the highest and lowest  values in the (OA)(AM)_2_ family is nearly 50%, the most significant variation within any family, followed by the (OA)(SA)_2_ family, where the highest Rayleigh intensity is approximately 11% greater than the lowest.

## 4 Conclusions

In the present work, an extensive DFT calculation, employing the M06 – 2*X*/6-311+ +G(3df,3pd) model, was performed on the hydrogen-boned molecular interactions between five stable structural conformers of OA (cTc, cTt, tTt, tCt, and cCt) and two important atmospheric nucleation precursor molecules, SA and AM. Several structural, thermodynamical, electrical, and spectroscopic parameters of the binary and ternary clusters mediated by oxalic acid were analyzed to gain insight into the hydrogen bonding nature of each OA conformer at standard atmospheric conditions. Multiple stable configurations for each kind of cluster composition, obtained by a combination of different quantum-chemical approaches and chemical intuition, were considered for the analysis. All OA conformers form strong hydrogen bonding with AM, showing thermodynamic stability at the ambient temperature, with average red shift of the OA O–H stretching mode of 934 (1088) cm^–1^ in binary (ternary) clusters. In ternary OA–AM clusters, the lowest energy OA conformer, cTc, has the lowest binding free energy, followed very closely by other conformers like tTt and tCt. In (OA)(SA) binary clusters, this same conformer exhibits the lowest binding energy. Although some of the binary (OA)(SA) clusters show positive values of binding free energy, Δ*G* at ambient temperature, the ternary (OA)(SA)_2_ clusters, however, show a different nature where all the conformations of each cluster composition show stability with negative Δ*G* values of varying magnitudes. Considering the effect of different conformations, the ternary OA–SA clusters can be arranged in the order of decreasing multiple-conformation binding free energy, Δ*G*_MC_ as follows: (cTt)(SA)_2_ >; (cTc)(SA)_2_ >; (tCt)(SA)_2_ >; (tTt)(SA)_2_ >; (cCt)(SA)_2_, with the highest energy OA conformer, cCt once again showing lowest free binding energy. Overall, the Δ*G* values of the (OA)(SA)_2_ clusters are much lower than those of the binary (OA)(SA) clusters, indicating higher stability for the ternary clusters. In general, OA–SA clusters have lower Δ*G* values than the OA-AM clusters according to the present calculations. Comparing the Δ*G*_S_ values for successive cluster formation for both (OA)(AM)_2_ and (OA)(SA)_2_, it is observed that the clusters of SA with OA are more likely to grow spontaneously. Consideration of partial pressures of the monomers in the calculation of binding free energy reveals that the Δ*G* values of the (OA)(AM)_n_ and (OA)(SA)_n_ clusters should be lower than −11.26 and −13.01 kcal/mol, respectively, in order to have a thermodynamical stability in a realistic atmospheric condition at 298.15K. Among all the clusters considered, only (cCt)(SA)_2_ satisfies this condition with Δ*G* = −13.31 kcal/mol. When comparing the Rayleigh activity of the clusters to that of the OA monomer, a notable increase in Rayleigh scattering intensity is observed due to the hydrogen-bonded molecular interactions present in all OA-mediated clusters. Specifically, the Rayleigh activity in the ternary (OA)(SA)_2_ clusters shows a variation exceeding 600% with respect to OA, which is the highest and significantly greater than that observed in all other clusters. The determination of excess Rayleigh activity due to clustering, calculated using a supermolecular approach, also shows a significant increase in all cases. Notably, the (OA)(SA)_2_ clusters exhibit considerably superior activity compared with other clusters. A less pronounced, but appreciable variation of Rayleigh activities is also observed when comparing the cluster among themselves, considering both size and composition. The average increase in Rayleigh scattering intensities observed going from binary to ternary clusters of OA, either with AM or SA, is close to 100%. Rayleigh intensities in (OA)(SA)_2_ clusters exceed those of (OA)(SA)_2_ clusters also by 100%, on average. The results obtained provide insights into the behavior of each stable structural conformer of oxalic acid, particularly in terms of their interaction potential with key atmospheric molecules under standard atmospheric conditions. We believe that this information may be relevant in the studies of environmental processes, given that oxalic acid is one of the most abundant naturally occurring dicarboxylic acids in the atmosphere.

## Supporting Information Available

Containing the optimized bond lengths and bond angles of different oxalic acid conformers; the Cartesian coordinates of the optimized geometries of binary and ternary clusters of OA with AM and SA; relevant structural parameters related to hydrogen bond formation in (OA)(SA) and (OA)(SA)_2_ clusters, supported by the ternary cluster images (Figure S1); successive binding free energies (Δ*G*_S_) for the formation of various (OA)(SA)_2_ ternary clusters and Boltzman-averaged values of Rayleigh scattering intensities of the OA conformers and their binary and ternatry clusters with AM and SA (PDF)
