# Supplementary material for: Intermolecular Hydrogen-Bonded Interactions of Oxalic Acid Conformers with Sulfuric Acid and Ammonia
Source: ACS Omega. 2024 Oct 1;9(41):42470–87. doi: 10.1021/acsomega.4c06290 (PMC11483408; doi:10.1021/acsomega.4c06290)
Supplement: Supplementary file 1 — ao4c06290_si_001.pdf [file ao4c06290_si_001.pdf]

SUPPORTING INFORMATION

Intermolecular Hydrogen-bonded interactions of Oxalic Acid conformers with Sulfuric Acid and Ammonia

Eduardo da Silva Carvalho, Angsula Ghosh, Puspitapallab Chaudhuri*

[1]Department of Materials Physics, Federal University of Amazonas, Manaus, AM, Brazil

TABLE S1: Optimized bond lengths, r (in Å) and bond angles, δ (in degrees) of different oxalic acid conformers, calculated by theoretical models - **M1:** M06-2X6-311++G(3df,3pd) – present work; **M2:** B3LYP/6-311++G(d,p) – Ref [62]; **M3:** MP2/6-311++G(d,p) – Ref [66]

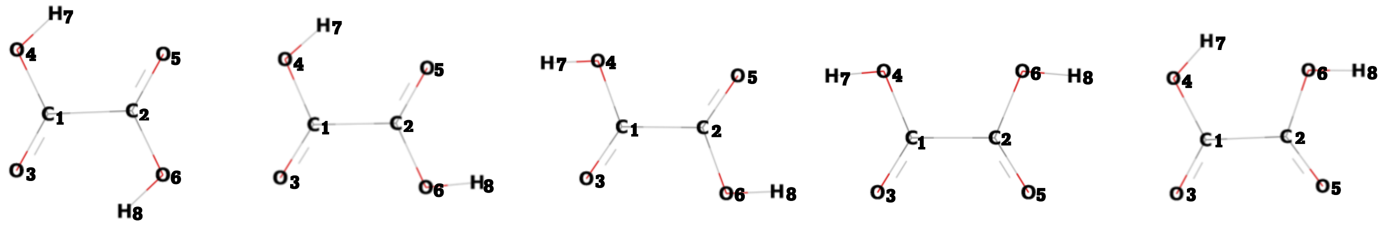

cTc **cTt** **tTt** **tCt** **cCt**

	cTc			cTt			tTt			tCt			cCt		
	**M1**	**M2**	**M3**	**M1**	**M2**	**M3**	**M1**	**M2**	**M3**	**M1**	**M2**	**M3**	**M1**	**M2**	**M3**
C_1-C_2	1.542	1.549	1.543	1.544	1.549	1.543	1.540	1.544	1.537	1.542	1.545	1.538	1.549	1.554	1.547
C_1-O_3	1.194	1.203	1.210	1.186	1.193	1.215	1.190	1.199	1.207	1.188	1.197	1.205	1.183	1.191	1.200
C_1-O_4	1.316	1.325	1.327	1.326	1.338	1.329	1.328	1.339	1.342	1.332	1.342	1.344	1.333	1.343	1.345
C_2-O_5	1.194	1.203	1.210	1.199	1.208	1.201	1.190	1.199	1.207	1.188	1.197	1.205	1.184	1.191	1.200
C_2-O_6	1.316	1.325	1.327	1.317	1.326	1.340	1.328	1.339	1.342	1.332	1.342	1.344	1.347	1.360	1.361
O_4-H_7 (O6-H8)	0.971	0.975	0.973	0.969	0.973	0.971	0.966	0.970	0.969	0.967	0.970	0.969	0.965	0.969	0.969
O_3-C_1-O_4	125.5	125.3	125.3	124.9	124.5	124.6	125.7	125.4	125.7	125.5	125.3	125.6	123.8	123.5	123.5
C_1-O_4-H_7	108.0	107.8	-	108.7	108.3	-	107.6	107.5	-	107.5	107.5	-	110.7	110.2	-
C_2-C_1-O_4	113.5	113.5	113.5	111.5	111.3	111.4	110.5	110.2	109.9	112.6	112.7	112.5	115.3	115.3	115.5
C_2-O_6-H_8	108.0	107.8	-	108.4	108.3	-	107.6	107.5	-	107.5	107.5	-	108.5	108.9	-

Gas phase experimental data, as reported in Ref [62]: r(O–H) =1.056, r(C–O) =1.339, r(C–C) =1.548, r(C=O) =1.208, δ(H-O-C) =104.4, δ(C-C=O) =123.1, δ(O-C=O) =125.0.

Table S2(a): The cartesian coordinates of the binary clusters (OA)(AM) cluster compositions. optimized at the MO6-2X/6-311++G(3df,3pd) level.

(cTc)(AM)

C	0.560507	0.809133	0.000200
C	0.658267	-0.742043	0.000141
O	1.910725	-1.153107	-0.000292
O	-0.287357	-1.472863	0.000261
O	1.594293	1.427357	0.000075
O	-0.622503	1.332662	-0.000107
H	2.468337	-0.354738	-0.000397
H	-1.423045	0.691124	-0.000138
N	-2.828467	-0.113453	-0.000126
H	-3.408195	0.028342	0.817853
H	-2.502994	-1.074022	-0.000306
H	-3.408749	0.028530	-0.817678

(cTt)(AM) – 1

C	0.015017	0.085577	0.000089
C	-1.508347	-0.186431	-0.000029
O	-2.205730	0.945093	0.000015
O	-1.988309	-1.271764	-0.000710
O	0.409635	1.231255	-0.000691
O	0.742528	-0.985535	0.000833
H	-1.572765	1.680208	0.000502
H	1.725641	-0.718026	0.000769
N	3.225983	-0.035000	0.000302
H	2.963189	0.945921	-0.000502
H	3.798736	-0.205815	-0.817371
H	3.798303	-0.204556	0.818542

(cTt)(AM) – 2

C	0.700134	-0.686503	0.000052
C	0.587974	0.859636	0.000085
O	-0.635378	1.320976	-0.000075
O	1.571458	1.534380	0.000074
O	-0.244308	-1.425596	0.000279
O	1.961105	-1.087380	-0.000238
H	-1.389571	0.649986	-0.000161
H	1.963368	-2.053705	-0.000217
N	-2.867132	-0.171139	-0.000127
H	-3.444730	-0.018944	0.817388
H	-2.565080	-1.138385	-0.000353
H	-3.445719	-0.018811	-0.816914

(tTt)(AM)

C	0.043251	0.123995	0.000060
C	-1.474587	-0.158352	-0.000135
O	0.733803	-0.984821	0.000252
O	0.480181	1.242680	-0.000025
O	-1.951096	-1.250180	-0.000291
O	-2.171050	0.976861	-0.000132
H	1.716801	-0.746510	0.000300
H	-3.106910	0.737230	-0.000279
N	3.240523	-0.035113	0.000223
H	2.916901	0.927917	0.000320
H	3.821208	-0.171490	-0.817900
H	3.821656	-0.171539	0.818016

(tCt)(AM)

C	0.044686	-0.200986	-0.000111
C	-1.494066	-0.061123	0.000081
O	-2.220523	-1.003730	0.000752
O	-1.897816	1.210465	-0.000607
O	0.546706	-1.289593	-0.000778
O	0.671885	0.948457	0.000547
H	1.668050	0.766367	0.000387
H	-2.864128	1.202911	-0.000478
N	3.222310	0.133452	0.000117
H	3.797347	0.293757	-0.817726
H	2.939412	-0.842616	-0.000140
H	3.797417	0.293280	0.818004

(cCt)(AM) – 1

C	0.013816	0.246927	-0.000011
C	-1.524317	0.049228	-0.000123
O	-1.917078	-1.225805	0.000034
O	-2.274244	0.966978	-0.000348
O	0.657906	-0.908623	-0.000038
O	0.516522	1.330712	0.000114
H	-1.138697	-1.798133	0.000226
H	1.664112	-0.726208	0.000046
N	3.178529	-0.119186	0.000223
H	3.749220	-0.294761	0.818348
H	2.924270	0.865073	0.000219
H	3.749545	-0.294701	-0.817688

(cCt)(AM) – 2

C	0.847997	0.708277	0.005619
C	0.691366	-0.834304	-0.022690
O	-0.529181	-1.318613	-0.018174
O	1.676292	-1.502125	-0.046996
O	-0.306777	1.361714	-0.230414
O	1.882060	1.255771	0.204401
H	-1.296510	-0.675885	0.023950
N	-2.864617	0.023398	0.100880
H	-3.009275	0.559010	-0.746721
H	-2.931422	0.668929	0.878556
H	-3.626242	-0.639486	0.182876
H	-0.099561	2.305827	-0.212922

Table S2(b): The cartesian coordinates of the ternary (OA)$(AM)_2$ cluster compositions. optimized at the MO6-2X/6-311++G(3df,3pd) level.

(cTc)$(AM)_2$– 1

C	0.015666	-0.778689	0.000118
C	-0.015652	0.778691	-0.000015
O	-1.195651	1.326318	0.000124
O	1.008882	1.408451	0.000296
O	-1.008872	-1.408443	-0.000161
O	1.195662	-1.326317	-0.000069
H	-1.997607	0.706515	0.000087
H	1.997635	-0.706520	-0.000059
N	3.507015	0.026381	-0.000095
H	4.084597	-0.123103	0.817559
H	3.192031	0.990318	-0.000054
H	4.084537	-0.123051	-0.817802
N	-3.507041	-0.026388	-0.000093
H	-3.192059	-0.990325	-0.000180
H	-4.084432	0.123177	-0.817869
H	-4.084761	0.122954	0.817489

(cTc)$(AM)_2$– 2

C	-1.353208	0.596113	0.228322
C	-0.778711	-0.743428	-0.315329
O	-1.675423	-1.708720	-0.283244
O	0.342538	-0.884270	-0.709972
O	-2.507495	0.581530	0.585704
O	-0.562034	1.608018	0.261577
H	-2.489215	-1.311458	0.078676
H	0.437661	1.486531	-0.084848
N	1.868376	1.520117	-0.542594
H	2.346756	2.407096	-0.441673
H	1.881028	1.265119	-1.523504
H	2.395866	0.796086	-0.045200
N	3.039543	-1.007869	0.726867
H	2.269388	-1.466347	0.249643
H	2.919968	-1.177261	1.718106
H	3.893946	-1.474075	0.448408

(cTt)$(AM)_2$– 1

C	0.374748	-0.296295	-0.000077
C	-0.894851	-1.189417	-0.000108
O	-2.034430	-0.538079	-0.000132
O	-0.804848	-2.378890	-0.000110
O	0.308702	0.912434	-0.000083
O	1.474815	-0.991993	0.000028
H	-1.984901	0.461300	-0.000133
H	2.261439	-0.361142	0.000078
N	-2.309142	2.170357	0.000149
H	-2.774948	2.543048	-0.817423
H	-1.349891	2.496306	0.000233
H	-2.774956	2.542693	0.817878
N	3.385081	0.905152	0.000174
H	2.712209	1.665144	0.000035
H	3.973242	1.005272	-0.817844
H	3.972946	1.005311	0.818401

(cTt)$(AM)_2$– 2

N	-2.483663	1.540393	0.001054
H	-2.818461	2.041598	-0.813486
H	-2.817819	2.038232	0.817912
H	-2.910242	0.606591	-0.000697
N	-3.203538	-1.403718	-0.001820
H	-2.193853	-1.522913	-0.002382
H	-3.569046	-1.884556	-0.814249
H	-3.567436	-1.881502	0.813145
C	0.517182	0.104386	0.000188
C	2.060834	-0.041030	-0.000245
O	2.431465	-1.318374	0.000905
O	2.815447	0.875225	-0.001725
O	-0.156300	-0.908965	0.001767
O	0.102378	1.319058	-0.000499
H	1.622048	-1.853231	0.001958
H	-0.946788	1.383364	-0.000081

(tTt)$(AM)_2$– 1

C	-0.763061	-0.124137	-0.000011
C	0.763056	0.124155	0.000155
O	-1.438239	0.999094	0.000161
O	-1.231401	-1.230858	0.000158
O	1.231396	1.230876	-0.000034
O	1.438232	-0.999076	-0.000049
H	-2.418240	0.776204	0.000236
H	2.418235	-0.776187	-0.000171
N	-3.967594	0.042474	-0.000184
H	-3.600045	-0.904886	-0.000512
H	-4.553618	0.153857	-0.818045
H	-4.553503	0.153300	0.817837
N	3.967606	-0.042514	-0.000105
H	3.600083	0.904856	-0.000033
H	4.553703	-0.153536	-0.817962
H	4.553434	-0.153733	0.817920

(tTt)$(AM)_2$– 2

N	-2.515740	1.530547	-0.000493
H	-2.860185	2.021785	-0.816873
H	-2.859436	2.026130	0.813582
H	-2.929120	0.591352	0.002053
N	-3.222227	-1.430590	0.002423
H	-2.210519	-1.536693	0.001337
H	-3.579752	-1.917032	-0.810286
H	-3.578130	-1.916757	0.816008
C	0.486892	0.071754	-0.001487
C	2.029058	-0.039829	0.000540
O	0.098259	1.307704	0.000204
O	-0.205580	-0.913865	-0.004272
O	2.769494	0.893840	0.002141
O	2.417874	-1.315085	0.000116
H	-0.936838	1.376273	-0.000497
H	3.383673	-1.317057	0.001337

$(tCt)(AM)_2$– 1

C	-0.771636	0.228513	-0.027097
C	0.771676	0.228464	0.027312
O	1.376663	1.243244	0.242070
O	1.299935	-0.953284	-0.191236
O	-1.376547	1.243316	-0.241987
O	-1.300006	-0.953149	0.191590
H	-2.301555	-0.861781	0.154974
H	2.301503	-0.862029	-0.154836
N	-3.925426	-0.343671	0.022015
H	-4.475927	-0.683355	-0.756696
H	-3.675470	0.623546	-0.165128
H	-4.511134	-0.364182	0.847555
N	3.925386	-0.343644	-0.022448
H	4.510347	-0.363714	-0.848527
H	4.476582	-0.683595	0.755641
H	3.675339	0.623433	0.165262

$(tCt)(AM)_2$– 2

N	-2.444448	-1.586193	-0.000191
H	-2.770421	-2.092587	0.814603
H	-2.770062	-2.091719	-0.815666
H	-2.895041	-0.663683	0.000189
N	-3.296342	1.335221	0.000763
H	-2.292066	1.497560	0.000234
H	-3.678131	1.802237	0.813901
H	-3.678950	1.802305	-0.811952
C	2.004065	0.267362	0.000137
C	0.476876	0.018188	-0.000472
O	-0.268763	0.961003	-0.001835
O	0.155597	-1.240314	0.000681
O	2.473986	1.361243	0.000641
O	2.715661	-0.861849	0.000014
H	3.648324	-0.608668	0.000403
H	-0.875620	-1.362596	0.000287

$(cCt)(AM)_2$– 1

C	0.491965	-0.066968	-0.000287
C	2.028410	-0.292519	0.000028
O	2.747576	0.831175	0.000410
O	2.507175	-1.377464	-0.000010
O	0.183196	1.207402	0.000143
O	-0.272000	-0.990057	-0.000735
H	2.143208	1.585583	0.000383
H	-0.864070	1.358224	0.000054
N	-3.304970	-1.293603	0.000482
H	-2.314298	-1.523796	-0.000319
H	-3.718531	-1.734628	-0.811836
H	-3.717026	-1.734091	0.813858
N	-2.374000	1.580170	-0.000221
H	-2.829620	0.657291	0.000001
H	-2.698216	2.086700	-0.815957
H	-2.698479	2.087229	0.815081

$(cCt)(AM)_2$– 2

C	0.330427	-0.670845	0.000079
C	-1.184439	-1.014627	-0.000087
O	-2.031192	-0.007234	-0.000376
O	-1.518630	-2.158216	-0.000010
O	1.164051	-1.529843	0.000564
O	0.583960	0.629817	-0.000370
N	-1.442114	2.652266	0.000346
H	-1.651033	0.914373	0.000044
H	1.586667	0.750355	-0.000383
H	-0.882721	2.874958	-0.813932
H	-0.882621	2.874981	0.814550
H	-2.254584	3.256330	0.000339
N	3.271447	0.583341	-0.000172
H	3.793570	0.874417	-0.817635
H	3.230553	-0.432915	0.000137
H	3.793391	0.874889	0.817242

Table S3(a): The cartesian coordinates of the binary (OA)(SA) cluster compositions, optimized at the MO6-2X/6-311++G(3df,3pd) level.

(cTc)(SA)– 1

C	-2.434046	-0.755379	-0.010665
C	-2.066084	0.749646	-0.030959
O	-3.112586	1.523727	0.089635
O	-0.942740	1.154370	-0.142162
O	-3.590095	-1.043531	0.124351
O	-1.457840	-1.602881	-0.140051
H	-3.893799	0.946059	0.168634
H	-0.569506	-1.197003	-0.252151
S	2.132352	-0.096829	-0.075612
O	1.147413	-1.025823	-0.519807
O	3.492579	-0.246840	-0.417022
O	1.709070	1.336689	-0.479171
O	1.985030	-0.084591	1.491932
H	0.733462	1.425833	-0.411025
H	2.766351	0.319815	1.892441

(cTc)(SA)– 2

C	-1.640549	0.738556	0.316044
C	-2.082378	-0.699586	-0.029856
O	-1.443719	-1.650196	0.606302
O	-2.972677	-0.864048	-0.809631
O	-0.846986	0.972312	1.190257
O	-2.228188	1.647641	-0.411960
H	-0.690090	-1.281348	1.094927
H	-2.830541	1.193001	-1.027268
S	1.741385	-0.027177	-0.281790
O	3.050223	-0.214896	-0.772454
O	0.593275	-0.109491	-1.110480
O	1.467065	-1.087956	0.870024
O	1.740641	1.313811	0.503535
H	2.281842	-1.264843	1.358894
H	0.837118	1.496781	0.840205

(cTc)(SA)– 3

C	1.489116	-0.570147	-0.574414
C	2.355987	0.336639	0.319104
O	2.495693	1.562368	-0.128310
O	2.844949	-0.102772	1.315028
O	1.043012	-0.185068	-1.625566
O	1.298186	-1.763733	-0.084536
H	1.966641	1.665852	-0.933899
H	1.743422	-1.808550	0.780074
S	-1.765020	0.235004	0.191835
O	-0.514568	0.666249	0.711913
O	-2.971985	0.913484	0.467374
O	-1.664430	0.102730	-1.355671
O	-1.902939	-1.262961	0.660951
H	-0.745178	-0.104416	-1.630961
H	-2.818529	-1.554280	0.557823

(cTt)(SA)– 1

C	-1.679982	-0.118873	-0.074036
C	-3.218964	-0.025074	0.045279
O	-1.228482	-1.322015	-0.113849
O	-1.026910	0.911086	-0.118003
O	-3.916923	-0.979904	0.106435
O	-3.646238	1.230432	0.070921
H	-0.237747	-1.328130	-0.182777
H	-2.886885	1.827342	0.007777
S	2.270568	-0.053751	-0.077681
O	1.406482	-1.172884	-0.299462
O	2.372585	0.052456	1.488520
O	1.535264	1.237051	-0.479040
O	3.566801	-0.019303	-0.631331
H	0.547656	1.143118	-0.336257
H	3.140909	0.586005	1.733171

(cTt)(SA)– 2

C	-2.489603	-0.790883	-0.004748
C	-2.142872	0.717882	-0.005058
O	-3.214288	1.474264	0.017188
O	-1.014436	1.138487	-0.013857
O	-3.611841	-1.151537	0.145006
O	-1.458717	-1.586667	-0.164422
H	-2.941737	2.402552	0.023122
H	-0.601056	-1.136323	-0.295729
S	2.155387	-0.072983	-0.103866
O	1.152845	-0.919300	-0.655610
O	3.488520	-0.096611	-0.565707
O	1.667465	1.398194	-0.169556
O	2.140343	-0.382680	1.439344
H	0.689068	1.423080	-0.109350
H	2.963253	-0.076781	1.843569

(cTt)(SA)– 3

C	1.755259	0.811445	-0.139645
C	2.024713	-0.703983	-0.155415
O	3.034739	-1.054572	0.610074
O	1.350749	-1.468714	-0.779515
O	2.324678	1.535274	0.622188
O	0.843531	1.127948	-1.030484
H	3.385740	-0.259371	1.042528
H	0.482116	2.013302	-0.872425
S	-1.868969	0.022828	0.109506
O	-1.739090	1.387361	-0.249408
O	-3.095219	-0.502283	0.574173
O	-1.388623	-0.913380	-1.034240
O	-0.743760	-0.217509	1.207377
H	-0.420035	-0.867413	-1.180861
H	-0.920183	-1.049528	1.667698

(tTt)(SA)– 1

C	1.655786	0.084882	-0.082642
C	3.191272	0.025557	0.043805
O	1.220783	1.302274	-0.129543
O	0.992753	-0.927732	-0.123680
O	3.876878	0.993383	0.123466
O	3.612828	-1.233284	0.051521
H	0.232141	1.316638	-0.194919
H	4.575861	-1.221103	0.132845
S	-2.280515	0.050196	-0.074815
O	-1.430567	1.181566	-0.290136
O	-2.363683	-0.074147	1.492884
O	-1.543459	-1.228416	-0.499331
O	-3.584505	0.014834	-0.612234
H	-0.550439	-1.138479	-0.348957
H	-3.099896	-0.650662	1.737522

(tTt)(SA)– 2

C	1.734590	0.865198	0.082172
C	1.984536	-0.650854	0.134403
O	0.889973	1.205080	1.050770
O	2.240077	1.591609	-0.707631
O	1.320211	-1.406671	0.791545
O	3.002575	-1.003052	-0.615942
H	0.638941	2.130988	0.932244
H	3.097887	-1.964988	-0.575521
S	-1.727680	-0.151221	-0.242669
O	-3.000902	-0.617288	-0.645989
O	-0.623521	-0.091595	-1.133993
O	-1.913413	1.325555	0.299701
O	-1.320525	-0.934807	1.029348
H	-2.826514	1.439087	0.592763
H	-0.338001	-1.022272	1.091298

(tTt)(SA)– 3

C	2.138117	0.830082	-0.007843
C	2.495712	-0.667672	-0.005698
O	0.816478	0.924960	-0.013609
O	2.926526	1.716718	-0.019933
O	1.661631	-1.519326	-0.121902
O	3.791152	-0.868117	0.123289
H	0.500288	1.836199	-0.092924
H	3.959090	-1.820497	0.104996
S	-2.203201	0.018139	-0.090623
O	-1.778328	1.286723	-0.558902
O	-2.004009	0.073951	1.476443
O	-1.203192	-1.097725	-0.513218
O	-3.492346	-0.487945	-0.373370
H	-0.267819	-0.857974	-0.356939
H	-2.486625	-0.656327	1.885689

(tCt)(SA)– 1

C	-1.647669	0.008694	-0.085272
C	-3.176113	0.174580	0.041731
O	-3.686242	1.245448	0.107353
O	-3.808253	-0.993782	0.065942
O	-0.955510	0.997271	-0.156233
O	-1.246910	-1.224113	-0.098213
H	-0.257853	-1.267968	-0.162459
H	-4.754997	-0.815162	0.146547
S	2.279544	-0.075679	-0.071157
O	1.400344	-1.191027	-0.253449
O	3.589077	-0.099300	-0.595330
O	1.584587	1.206284	-0.550942
O	2.348842	0.103547	1.491861
H	0.588805	1.156197	-0.395932
H	3.086553	0.683532	1.723679

(tCt)(SA)– 2

C	1.696959	0.784614	0.230308
C	1.882263	-0.739675	0.168359
O	1.484712	-1.430538	1.051883
O	2.509859	-1.137959	-0.925177
O	0.964420	1.282005	1.039736
O	2.419164	1.450538	-0.645292
H	2.224864	2.393509	-0.547953
H	2.541968	-2.104360	-0.917350
S	-1.704486	-0.155444	-0.190083
O	-0.550180	-0.352802	-0.996043
O	-2.868548	-0.942818	-0.341358
O	-1.320815	-0.204749	1.312005
O	-2.083825	1.362287	-0.404665
H	-0.433940	0.197665	1.453227
H	-2.974756	1.522938	-0.067313

(tCt)(SA)– 3

C	2.192606	0.818736	-0.025486
C	2.838622	-0.579896	0.025365
O	4.002636	-0.745187	0.175273
O	1.927607	-1.549897	-0.121959
O	2.819628	1.820707	0.039523
O	0.866575	0.727169	-0.150117
H	0.434383	1.594845	-0.199880
H	2.385738	-2.400766	-0.089525
S	-2.275606	0.027565	-0.082819
O	-1.760550	1.306432	-0.421137
O	-3.523707	-0.435249	-0.554476
O	-1.228497	-1.071015	-0.441171
O	-2.281714	-0.005959	1.493563
H	-0.320832	-0.722015	-0.342062
H	-2.852766	-0.722153	1.801306

(cCt)(SA)– 1

S	-2.257221	0.073860	-0.066870
O	-1.372319	1.168476	-0.339623
O	-3.590057	0.101352	-0.524583
O	-1.606715	-1.236523	-0.533003
O	-2.239849	-0.041359	1.502234
H	-0.610342	-1.209325	-0.395863
H	-2.962849	-0.609104	1.802006
C	1.644441	-0.059022	-0.099603
C	3.181515	-0.175349	0.050642
O	3.823947	0.986992	0.109543
O	3.710255	-1.232671	0.107190
O	1.234446	1.182245	-0.146282
O	0.951527	-1.042471	-0.158078
H	3.201982	1.722696	0.043839
H	0.241134	1.231868	-0.225478

(cCt)(SA)– 2

C	-3.000753	-0.496586	0.008888
C	-2.161065	0.801955	0.001602
O	-0.838090	0.615970	-0.066363
O	-2.665076	1.869910	0.052811
O	-2.209649	-1.577257	-0.037936
O	-4.184090	-0.507854	0.050349
H	-0.573602	-0.321164	-0.113263
H	-2.759022	-2.372501	-0.035614
S	2.296324	-0.157012	-0.089911
O	3.568790	-0.339718	-0.671769
O	1.236261	-1.087267	-0.277436
O	2.455310	-0.036991	1.470988
O	1.812769	1.266604	-0.498022
H	3.299867	0.381827	1.684698
H	0.852681	1.364638	-0.341151

(cCt)(SA)– 3

C	1.959504	0.802483	-0.042020
C	2.410181	-0.667575	0.004542
O	1.484251	-1.568010	-0.288088
O	3.525179	-0.933496	0.312345
O	0.910402	1.023672	0.792504
O	2.471719	1.654499	-0.684222
H	0.655667	-1.181224	-0.628291
H	0.668785	1.959111	0.717643
S	-1.928234	-0.163976	-0.117976
O	-0.913767	-0.456606	-1.074058
O	-3.222066	-0.714234	-0.207422
O	-1.391447	-0.477424	1.309085
O	-2.028956	1.409314	-0.109878
H	-0.477693	-0.143165	1.395745
H	-2.895650	1.677730	0.225251

Table S3(b): The cartesian coordinates of the ternary (OA)$(SA)_2$ cluster compositions, optimized at the MO6-2X/6-311++G(3df,3pd) level.

(cTc)$(SA)_2$– 1

S	0.015412	2.129001	-0.073749
O	-0.958586	1.608585	-0.986190
O	1.376066	2.181532	-0.475462
O	-0.343790	3.611420	0.261879
O	-0.122324	1.428658	1.276045
H	-1.282859	3.705345	0.472081
H	-0.751473	0.620971	1.231976
S	-2.440314	-1.174412	0.130603
O	-3.468411	-2.067204	0.474283
O	-1.690068	-0.533939	1.191187
O	-1.428774	-1.883048	-0.792444
O	-3.058417	-0.067567	-0.762324
H	-0.512793	-1.506325	-0.693800
H	-2.384086	0.617363	-0.963750
C	1.937605	-1.454287	-0.181893
C	3.325128	-0.820325	0.073175
O	3.404729	0.473237	-0.002402
O	4.232319	-1.563399	0.320233
O	0.956519	-0.804388	-0.445353
O	1.943556	-2.754050	-0.087892
H	2.557685	0.928681	-0.208130
H	2.853006	-3.030471	0.131778

(cTc)$(SA)_2$– 2

C	-0.185471	-0.752911	-0.386588
C	0.185129	0.752650	-0.386667
O	-0.804252	1.584991	-0.389676
O	1.341555	1.085867	-0.383182
O	-1.341879	-1.086177	-0.383191
O	0.803957	-1.585204	-0.389347
H	-1.699109	1.169952	-0.388014
H	1.698757	-1.170063	-0.387521
S	4.362486	-0.101556	0.097197
O	3.431444	-1.062533	-0.394123
O	5.752035	-0.273657	-0.072590
O	3.999573	1.300357	-0.448941
O	4.032732	0.016527	1.632244
H	3.022864	1.399001	-0.491157
H	4.759691	0.458738	2.091375
S	-4.362400	0.101620	0.097211
O	-5.752063	0.273664	-0.071688
O	-3.431669	1.062215	-0.395452
O	-4.031511	-0.015435	1.632090
O	-3.999933	-1.300675	-0.448246
H	-4.758171	-0.457231	2.092094
H	-3.023253	-1.399373	-0.490973

(cTc)$(SA)_2$– 3

S	-2.346696	-1.150196	0.106807
O	-0.922849	-1.139548	-0.054357
O	-3.049447	-2.350109	0.304764
O	-2.670573	-0.192417	1.301110
O	-2.985790	-0.432355	-1.112904
H	-1.947046	0.447468	1.416663
H	-2.503716	0.399115	-1.290875
S	-0.087791	1.846825	-0.302421
O	0.979165	1.187220	-0.951496
O	-1.414535	1.832074	-0.833216
O	-0.190374	1.369868	1.175405
O	0.333138	3.343450	-0.144187
H	0.514560	0.679649	1.361421
H	-0.441365	3.906600	-0.010704
C	2.641731	-0.579416	0.749486
C	2.655000	-1.393859	-0.568740
O	1.521566	-1.867286	-0.987656
O	3.716642	-1.557637	-1.102299
O	1.655456	-0.403156	1.418571
O	3.823054	-0.132619	1.069726
H	0.743070	-1.563648	-0.477417
H	4.442295	-0.435476	0.378568

(cTc)$(SA)_2$– 4

C	-0.506084	0.576520	1.107713
C	-0.098183	1.290515	-0.202641
O	-1.023572	2.047564	-0.719944
O	1.002498	1.170166	-0.664675
O	-1.561813	0.860901	1.622839
O	0.327640	-0.282215	1.587610
H	-1.861374	1.929819	-0.238851
H	1.150722	-0.414017	1.057933
S	-3.470970	-0.639904	-0.351475
O	-4.521325	-1.360504	-0.956742
O	-2.112688	-0.804934	-0.725718
O	-3.739184	0.916318	-0.545799
O	-3.629772	-0.808360	1.184296
H	-4.688256	1.096455	-0.515398
H	-2.903414	-0.332165	1.643188
S	3.795683	-0.483378	-0.136776
O	2.673815	-1.023254	0.557961
O	4.933989	-1.269303	-0.411101
O	3.330230	0.138545	-1.475467
O	4.217067	0.771771	0.713998
H	2.456866	0.571850	-1.356687
H	5.100582	1.064802	0.453350

(cTc)$(SA)_2$– 5

C	3.105692	0.206776	-0.350947
C	2.860309	-1.158210	0.334441
O	3.959092	-1.841057	0.521349
O	1.767704	-1.541583	0.646662
O	4.200233	0.469216	-0.752107
O	2.055046	0.979431	-0.452665
H	4.702025	-1.327104	0.160106
H	1.260135	0.659109	0.048567
S	-0.924052	2.146688	0.245413
O	-0.245351	0.970199	0.732285
O	-1.793741	2.839483	1.106832
O	0.134647	3.171606	-0.264165
O	-1.607205	1.775333	-1.093584
H	0.893095	2.731872	-0.681193
H	-2.069792	0.900938	-0.994139
S	-1.914288	-1.637579	-0.024335
O	-1.781883	-1.743576	1.372424
O	-2.684270	-0.586659	-0.628535
O	-0.525817	-1.628518	-0.711400
O	-2.529801	-2.981101	-0.542233
H	0.213052	-1.618387	-0.058956
H	-2.970330	-2.845779	-1.391496

(cTt)(SA)$_2$– 1

C	0.431051	1.596996	0.272108
C	1.872164	2.105440	0.074001
O	1.975178	3.034290	-0.845493
O	2.793187	1.686107	0.711453
O	-0.440232	1.987558	-0.480251
O	0.313242	0.795999	1.272613
H	1.106288	3.174468	-1.253272
H	-0.597584	0.391807	1.312520
S	1.928062	-1.525634	-0.252861
O	2.516374	-2.698506	-0.778287
O	1.550614	-0.433041	-1.083906
O	0.606396	-1.936143	0.504798
O	2.841027	-1.013588	0.888929
H	0.695230	-2.832695	0.853564
H	2.774654	-0.034678	0.999192
S	-2.986302	-0.215444	0.089656
O	-2.119088	-0.189301	1.228974
O	-4.342815	-0.572630	0.229251
O	-2.918519	1.143835	-0.632315
O	-2.275695	-1.197397	-0.909124
H	-1.990535	1.513947	-0.596551
H	-2.872839	-1.427674	-1.633978

(cTt)(SA)$_2$– 2

S	-2.215985	-1.489129	0.069498
O	-0.916501	-1.187989	-0.464867
O	-2.657323	-2.816434	0.188315
O	-2.295891	-0.822317	1.478872
O	-3.263941	-0.713159	-0.767444
H	-1.704071	-0.048050	1.521234
H	-2.960554	0.209163	-0.897411
S	-0.625981	1.903601	-0.243819
O	0.354829	1.538543	-1.190686
O	-2.022976	1.723134	-0.495303
O	-0.328204	1.212659	1.113985
O	-0.406395	3.418604	0.077111
H	0.574070	0.721288	1.069572
H	-1.203642	3.807433	0.461730
C	2.367739	-0.607782	0.053193
C	3.902610	-0.751564	-0.074013
O	4.555370	-0.051138	0.844129
O	4.413729	-1.420903	-0.905940
O	1.918285	0.088148	0.954765
O	1.710668	-1.266698	-0.825104
H	3.919365	0.409969	1.408706
H	0.730991	-1.194874	-0.712441

(cTt)(SA)$_2$– 3

C	0.645383	2.393003	-0.409833
C	1.527297	1.823876	0.718806
O	0.871693	1.042194	1.573749
O	2.682709	2.069908	0.807236
O	-0.549038	2.257249	-0.432618
O	1.351974	3.032208	-1.308502
H	-0.057132	0.829927	1.306118
H	0.761344	3.361002	-2.000846
S	-2.437226	-0.268993	0.145337
O	-1.393384	-0.190021	1.131962
O	-3.508523	-1.160802	0.329774
O	-3.053952	1.146905	-0.045806
O	-1.769622	-0.536178	-1.231641
H	-2.337281	1.793403	-0.176012
H	-0.789406	-0.507092	-1.162000
S	1.486845	-1.724685	-0.290264
O	0.932574	-0.542477	-0.887254
O	2.441111	-2.507752	-0.969792
O	2.069606	-1.360773	1.091980
O	0.232092	-2.600864	0.047154
H	1.648755	-0.552195	1.465658
H	0.485814	-3.524257	0.182123

(cTt)(SA)$_2$– 4

S	0.132966	2.226116	-0.019095
O	-0.561754	1.033627	-0.430522
O	-0.554528	3.447368	0.080449
O	0.795597	1.924962	1.361422
O	1.342228	2.440547	-0.964748
H	0.993212	0.974507	1.450825
H	1.868342	1.617894	-1.032785
S	2.005373	-1.199997	-0.085679
O	1.238757	-1.993277	-0.987020
O	2.595948	0.020945	-0.537507
O	1.191995	-0.913361	1.175654
O	3.155957	-2.124248	0.416219
H	0.163640	-1.241500	1.099040
H	3.905660	-1.603764	0.736073
C	-1.834424	-1.344503	0.011497
C	-3.264637	-0.802748	0.049876
O	-3.351009	0.491640	-0.204521
O	-4.183150	-1.512092	0.299942
O	-1.177021	-1.423319	1.039927
O	-1.441159	-1.673685	-1.173878
H	-2.475898	0.888290	-0.364378
H	-0.488890	-1.922695	-1.183978

(cTt)(SA)$_2$– 5

C	-0.750622	1.432937	-0.101306
C	0.588939	2.188337	0.038123
O	-1.784432	2.195370	-0.023746
O	-0.731631	0.227650	-0.264009
O	0.669349	3.355725	0.212355
O	1.634499	1.369616	-0.058388
H	-2.620004	1.664815	-0.115300
H	1.368463	0.440512	-0.204269
S	-3.990288	-0.770945	-0.061160
O	-3.900376	0.639543	-0.287279
O	-3.848680	-0.917241	1.499097
O	-2.721347	-1.454143	-0.602207
O	-5.110878	-1.503172	-0.502705
H	-1.927553	-0.857797	-0.491975
H	-4.130731	-1.799376	1.776590
S	3.942096	-0.902177	-0.091434
O	4.980328	-1.658282	-0.675872
O	2.571981	-1.128352	-0.398528
O	4.031785	-1.028874	1.474723
O	4.284513	0.598844	-0.334926
H	4.953756	-1.138311	1.743472
H	3.496363	1.159012	-0.196021

(tTt)$(SA)_2$– 1

C	-0.770446	0.044296	-0.326583
C	0.770496	-0.044221	-0.326632
O	-1.188487	1.268373	-0.316835
O	-1.444377	-0.959578	-0.332340
O	1.444429	0.959650	-0.332373
O	1.188530	-1.268301	-0.316975
S	-4.672842	0.067348	0.062458
O	-3.833653	1.177098	-0.273650
O	-4.552024	-0.042910	1.628248
O	-4.024755	-1.232150	-0.440795
O	-6.035077	0.057684	-0.302557
S	4.672835	-0.067358	0.062458
O	4.551735	0.042504	1.628254
O	3.833696	-1.177016	-0.274079
O	6.035135	-0.057617	-0.302310
O	4.024843	1.232274	-0.440580
H	-2.180588	1.296176	-0.304704
H	2.180633	-1.296111	-0.304918
H	-3.022600	-1.167134	-0.405629
H	3.022689	1.167269	-0.405529
H	5.260614	0.600789	1.974822
H	-5.260911	-0.601359	1.974537

(tTt)$(SA)_2$– 2

C	-1.804516	2.252279	-0.051396
C	-0.365053	1.785200	0.233805
O	-2.644297	1.694433	0.820471
O	-2.094219	3.004507	-0.920494
O	-0.150317	0.918793	1.068994
O	0.506926	2.396021	-0.477608
H	-3.542171	1.964863	0.586883
H	1.431660	2.032313	-0.333340
S	-1.529400	-1.603991	-0.188479
O	-0.247219	-2.243922	-0.228912
O	-1.802514	-0.541158	-1.081810
O	-2.615738	-2.703139	-0.386166
O	-1.852817	-1.181668	1.267331
H	-2.275401	-3.568260	-0.120113
H	-1.452085	-0.296141	1.431587
S	2.752789	-0.151764	-0.029994
O	2.844372	1.281358	-0.107478
O	3.910988	-0.933333	-0.196211
O	2.107703	-0.550431	1.327316
O	1.642185	-0.510076	-1.060323
H	1.305055	0.001934	1.461352
H	1.135702	-1.318578	-0.826120

(tTt)$(SA)_2$– 3

S	-2.211405	-1.502385	0.064612
O	-0.927963	-1.195600	-0.502968
O	-2.650538	-2.831572	0.178182
O	-2.253401	-0.855075	1.484433
O	-3.282569	-0.715699	-0.732393
H	-1.665103	-0.076652	1.518590
H	-2.980304	0.208287	-0.858565
S	-0.650872	1.902386	-0.236662
O	0.321606	1.556177	-1.199706
O	-2.049629	1.716995	-0.480823
O	-0.338338	1.202011	1.107758
O	-0.439427	3.418015	0.093000
H	0.586385	0.726308	1.063341
H	-1.228454	3.790195	0.509075
C	2.363111	-0.562047	0.078241
C	3.888402	-0.736372	-0.064070
O	1.718227	-1.226544	-0.814435
O	1.903161	0.129936	0.965656
O	4.387704	-1.445164	-0.876421
O	4.530847	0.001404	0.832964
H	0.738861	-1.156288	-0.712858
H	5.478526	-0.140446	0.706206

(tTt)$(SA)_2$– 4

C	0.487139	1.632607	0.232158
C	1.941311	2.103540	0.062276
O	0.373116	0.842434	1.252857
O	-0.379554	1.996927	-0.523371
O	2.865274	1.566683	0.607879
O	2.031124	3.142246	-0.736418
H	-0.537277	0.443088	1.294439
H	2.964177	3.378183	-0.835199
S	1.804520	-1.627704	-0.246678
O	1.492191	-0.537812	-1.105328
O	0.474504	-1.920031	0.548143
O	2.774362	-1.142089	0.862740
O	2.303924	-2.855434	-0.739378
H	2.750777	-0.160675	0.946623
H	0.500917	-2.812736	0.916059
S	-2.959704	-0.131875	0.090163
O	-4.326394	-0.444341	0.245786
O	-2.083316	-0.126765	1.222472
O	-2.292475	-1.150632	-0.903668
O	-2.853126	1.213182	-0.647550
H	-2.893727	-1.343967	-1.635660
H	-1.909653	1.557526	-0.621932

(tTt)$(SA)_2$– 5

C	2.403182	-0.159534	0.005179
C	3.941917	-0.250823	0.040932
O	1.856229	-1.078978	0.741132
O	1.841191	0.692031	-0.639962
O	4.531535	-1.019097	0.730184
O	4.480102	0.635643	-0.785338
H	0.875549	-1.046129	0.718325
H	5.441175	0.547285	-0.731142
S	-1.577186	-1.707918	-0.176047
O	-0.905701	-1.086344	0.953397
O	-0.797300	-1.966093	-1.320364
O	-2.142997	-3.084313	0.293919
O	-2.864158	-0.943644	-0.458843
H	-2.704275	-2.985021	1.075154
H	-2.804096	0.062414	-0.242635
S	-1.321814	2.030693	0.085969
O	-1.151598	3.418322	-0.061396
O	-2.663454	1.494170	0.146761
O	-0.638138	1.208023	-1.020533
O	-0.559855	1.605401	1.380990
H	0.350420	1.059706	-0.885081
H	-0.732240	0.658521	1.550384

(tCt)$(SA)_2$– 1

S	-1.495728	-1.604320	-0.156122
O	-0.227941	-2.158710	0.218819
O	-1.551783	-0.599021	-1.154413
O	-2.391352	-2.794473	-0.635072
O	-2.240361	-1.145824	1.109418
H	-2.086153	-3.623362	-0.242668
H	-2.410151	-0.162712	1.079226
S	2.791381	-0.086836	-0.032228
O	2.733187	1.327549	-0.299028
O	4.029580	-0.746740	-0.144695
O	2.222552	-0.371510	1.381759
O	1.712923	-0.705544	-0.968333
H	1.363285	0.105778	1.478401
H	1.164976	-1.395647	-0.532646
C	-1.910060	2.062683	0.118090
C	-0.418263	1.694849	0.227808
O	-0.101243	0.915077	1.096471
O	0.337068	2.275475	-0.637030
O	-2.752238	1.421796	0.689205
O	-2.143520	3.124590	-0.614550
H	-3.097599	3.287167	-0.639289
H	1.290173	1.960754	-0.545227

(tCt)$(SA)_2$– 2

C	0.768754	0.237682	0.070595
C	-0.768794	0.237703	-0.069848
O	-1.351314	-0.819159	-0.129953
O	-1.289157	1.422115	-0.107415
O	1.351211	-0.819206	0.130960
O	1.289196	1.422067	0.107776
H	2.277310	1.364959	0.189747
H	-2.277263	1.365044	-0.189426
S	4.684750	-0.070784	0.086772
O	3.906346	1.106773	0.327151
O	5.983965	-0.197291	0.620812
O	3.870069	-1.309346	0.489401
O	4.755899	-0.168512	-1.482563
H	2.886369	-1.153938	0.348956
H	5.456777	-0.780941	-1.743199
S	-4.684745	-0.070736	-0.087071
O	-3.906345	1.106902	-0.327039
O	-5.983927	-0.197103	-0.621217
O	-3.870053	-1.309190	-0.490075
O	-4.755909	-0.168956	1.482230
H	-2.886436	-1.153982	-0.348990
H	-5.456435	-0.781875	1.742666

(tCt)$(SA)_2$– 3

S	-1.366604	-1.777719	0.051678
O	-1.078377	-1.034696	1.244312
O	-0.310654	-1.947990	-0.889968
O	-1.791132	-3.228609	0.435632
O	-2.633537	-1.250228	-0.601076
H	-2.515068	-3.219323	1.077028
H	-2.780056	-0.237396	-0.417810
S	-1.635853	1.909605	-0.033877
O	-1.649344	3.291850	-0.287795
O	-2.891135	1.187603	-0.085962
O	-0.719209	1.117328	-0.987468
O	-0.983939	1.693797	1.359315
H	0.256742	1.101344	-0.735922
H	-0.975253	0.735742	1.567092
C	2.509102	-0.014259	-0.243887
C	3.983637	0.268711	0.115050
O	4.367434	1.365521	0.359682
O	4.723566	-0.835203	0.111090
O	1.742084	0.913805	-0.325933
O	2.246015	-1.272192	-0.421796
H	1.294271	-1.441260	-0.625226
H	5.628055	-0.583892	0.342772

(tCt)$(SA)_2$– 4

S	-2.171224	-1.537831	0.055607
O	-0.889173	-1.203649	-0.500446
O	-2.580930	-2.876793	0.166039
O	-2.241360	-0.890937	1.474017
O	-3.251035	-0.775915	-0.753074
H	-1.675116	-0.096249	1.512522
H	-2.967278	0.154764	-0.877317
S	-0.691320	1.901237	-0.241795
O	0.302525	1.568838	-1.187864
O	-2.081604	1.681801	-0.505756
O	-0.385600	1.218958	1.113109
O	-0.517366	3.423601	0.076299
H	0.554537	0.771007	1.092573
H	-1.311516	3.778344	0.497914
C	2.374724	-0.481117	0.152657
C	3.912555	-0.580438	0.084157
O	4.610492	0.018912	0.835530
O	4.318279	-1.391616	-0.884713
O	1.882013	0.203690	1.024863
O	1.760553	-1.167647	-0.748859
H	0.777135	-1.119948	-0.666456
H	5.284903	-1.407019	-0.874284

(tCt)$(SA)_2$– 5

C	-1.838470	-1.036014	-0.219728
C	-3.296412	-0.665022	0.106007
O	-3.604298	0.471726	0.285062
O	-4.104276	-1.712737	0.156563
O	-1.022752	-0.151883	-0.325667
O	-1.605224	-2.301655	-0.352754
H	-0.639735	-2.430074	-0.553995
H	-4.995170	-1.397233	0.361766
S	0.498345	2.616922	-0.125144
O	1.079834	1.705699	-1.059457
O	0.758813	4.001588	-0.205043
O	-1.023789	2.421452	-0.076518
O	0.982035	2.080107	1.282629
H	-1.249499	1.448985	-0.157167
H	0.847745	2.766780	1.949584
S	1.977292	-1.805047	0.004232
O	0.985916	-2.381854	-0.849201
O	3.124700	-2.546905	0.356984
O	2.462830	-0.457735	-0.545060
O	1.175987	-1.409597	1.304025
H	1.767319	0.222407	-0.755945
H	1.790229	-1.140284	2.000162

(cCt)(SA)$_2$– 1

S	1.317953	1.640193	-0.161952
O	-0.016374	1.990633	0.221305
O	1.523015	0.632516	-1.139448
O	2.004817	2.941472	-0.687217
O	2.136854	1.342711	1.103508
H	1.606074	3.723938	-0.283318
H	2.454985	0.393998	1.126240
S	-2.867266	-0.143913	-0.036981
O	-2.072318	-0.945175	-0.938293
O	-4.261900	-0.316116	-0.021131
O	-2.311969	-0.353918	1.393437
O	-2.585572	1.347242	-0.335428
H	-1.345793	-0.568939	1.364505
H	-1.634490	1.574478	-0.230337
C	0.753005	-1.617587	0.207013
C	2.280537	-1.821629	0.165571
O	2.716012	-2.769548	-0.628570
O	3.003674	-1.138874	0.835102
O	0.139254	-2.198674	-0.777873
O	0.265324	-0.962847	1.093915
H	1.981676	-3.146038	-1.134933
H	-0.801225	-1.838016	-0.849188

(cCt)(SA)$_2$– 2

S	1.327696	1.793202	0.051519
O	1.111592	1.044977	1.255346
O	0.222166	1.939778	-0.839002
O	1.732474	3.254705	0.411812
O	2.569993	1.294216	-0.663891
H	2.492905	3.268422	1.009926
H	2.755848	0.286038	-0.478131
S	1.691933	-1.887605	-0.037825
O	1.736000	-3.268251	-0.293135
O	2.919942	-1.125608	-0.140635
O	0.715662	-1.121773	-0.956925
O	1.087281	-1.689006	1.378176
H	-0.249652	-1.156948	-0.685422
H	1.064727	-0.732416	1.590296
C	-2.538455	-0.068593	-0.208351
C	-4.033839	-0.321205	0.113010
O	-4.793536	0.769945	0.130435
O	-4.433999	-1.414796	0.322020
O	-2.278951	1.201100	-0.396619
O	-1.757951	-0.981135	-0.262578
H	-4.266155	1.554521	-0.065522
H	-1.323350	1.396407	-0.578229

(cCt)(SA)$_2$– 3

S	-2.156696	-1.534301	0.058865
O	-0.872882	-1.195590	-0.494760
O	-2.558347	-2.875220	0.168071
O	-2.232910	-0.887115	1.475446
O	-3.233047	-0.777467	-0.756588
H	-1.678763	-0.084032	1.515564
H	-2.952170	0.153803	-0.884348
S	-0.678979	1.905595	-0.246592
O	0.323817	1.552351	-1.175278
O	-2.066749	1.683331	-0.518686
O	-0.391035	1.241406	1.124061
O	-0.501106	3.430017	0.049068
H	0.546478	0.805497	1.125964
H	-1.294713	3.797002	0.461715
C	2.379357	-0.468073	0.197371
C	3.918342	-0.593745	0.075371
O	4.336501	-1.359789	-0.925671
O	4.634537	-0.035172	0.834133
O	1.746354	-1.172799	-0.693816
O	1.892168	0.222140	1.061286
H	3.586878	-1.716930	-1.418626
H	0.758486	-1.133875	-0.611200

(cCt)(SA)$_2$– 4

S	2.769088	0.194984	-0.099909
O	1.954332	0.253726	-1.274964
O	4.166397	0.040003	-0.187815
O	2.441085	1.348186	0.832943
O	2.171840	-1.079667	0.660199
H	1.467861	1.672488	0.725486
H	2.718073	-1.320277	1.420791
C	-0.808758	1.728905	-0.165241
C	-2.286471	2.100161	0.081998
O	-3.181148	1.274521	-0.434317
O	-2.556173	3.083367	0.688697
O	-0.632082	1.018776	-1.248840
O	0.056583	2.105082	0.588930
H	-2.789697	0.479079	-0.830107
H	0.314592	0.766101	-1.362034
S	-1.321031	-1.720114	0.107934
O	-2.373812	-1.482194	-0.816600
O	-0.974410	-0.764419	1.095672
O	-1.623969	-3.053416	0.873927
O	-0.064044	-2.076033	-0.734468
H	-2.140752	-3.652304	0.317427
H	0.755600	-1.860853	-0.247415

(cCt)(SA)$_2$– 5

S	2.748553	-0.485692	0.100693
O	1.806003	-0.484231	1.180319
O	4.072874	-0.925958	0.297207
O	2.137586	-1.179082	-1.110098
O	2.815355	1.039316	-0.349282
H	1.139168	-1.377758	-1.010522
H	3.660173	1.218334	-0.783904
C	-1.102475	-1.543363	0.010257
C	-2.556446	-2.038334	-0.033203
O	-3.494712	-1.115446	-0.108158
O	-2.758790	-3.207549	0.025996
O	-0.795508	-0.920895	1.129239
O	-0.321941	-1.779772	-0.880716
H	-3.129934	-0.220613	-0.235366
H	0.185342	-0.752014	1.160002
S	-1.133424	2.058418	-0.113327
O	-2.252412	1.282727	-0.532496
O	-1.019619	3.426747	-0.420926
O	-0.978694	1.931518	1.434988
O	0.102147	1.269963	-0.664618
H	-1.185049	1.024301	1.717328
H	0.943423	1.735626	-0.519358

Table S4: Relevant structural parameters related to hydrogen bond formation in (OA)(SA) binary clusters obtained at the MO6-2X/6-311++G(3df,3pd) level of calculation.

	PD*	R_{O-O} (Å)	$R_{(O)H\cdots O}$ (Å)	$\angle O-H\cdots O$ (degrees)	ΔR_{O-H} (Å)	ν_{O-H} (cm^{-1})	$\Delta\nu_{O-H}$ (cm^{-1})
(cTc)(SA) −1	OA	2.695	1.746	161.2	0.012	3482	-246
	SA	2.679	1.719	164.8	0.016	3533	-288
(cTc)(SA) −2	SA	2.699	1.798	150.9	0.015	3520	-301
(cTc)(SA) −3	SA	2.736	1.790	160.8	0.016	3511	-310
(cTt)(SA) −1	OA	2.646	1.656	174.3	0.026	3269	-551
	SA	2.608	1.606	177.1	0.036	3065	-756
(cTt)(SA) −2	OA	2.709	2.052	122.8	0.008	3649	-124
	SA	2.768	1.866	152.1	0.011	3604	-217
(cTt)(SA) −3	SA	2.807	1.913	150.3	0.014	3561	-260
(tTt)(SA) −1	OA	2.659	1.671	174.5	0.024	3301	-531
	SA	2.581	1.573	177.4	0.042	2975	-846
(tTt)(SA) −2	SA	2.693	1.728	164.3	0.022	3359	-462
(tTt)(SA) −3	OA	2.676	2.390	96.4	0.001	3812	-20
	SA	2.922	2.053	146.9	0.012	3629	-192
(tCt)(SA) −1	OA	2.652	1.662	174.8	0.025	3280	-541
	SA	2.579	1.571	177.0	0.043	2971	-850
(tCt)(SA) −2	SA	2.740	1.817	154.8	0.018	3501	-320
(tCt)(SA) −3	OA	2.704	2.225	109.2	0.004	3760	-61
	SA	2.776	1.883	150.5	0.012	3624	-197
(cCt)(SA) −1	OA	2.614	1.619	174.9	0.031	3173	-651
	SA	2.592	1.589	175.4	0.040	3002	-819
(cCt)(SA) −2	OA	2.716	1.857	144.5	0.014	3553	-271
	SA	2.929	2.048	149.6	0.007	3708	-113
(cCt)(SA) −3	OA	2.692	1.972	128.8	0.010	3679	-151
	SA	2.763	1.869	150.6	0.012	3597	-224

*PD: the proton-donor monomer for hydrogen bond (HB) formation – OA: Oxalic Acid donating proton via its OH bond; SA: Sulfuric acid donating proton donor via its OH bond.

R_{O-O}: Distance between the oxygen atom of OA and that of SA forming the $O-H\cdots O$ HB.

$R_{(O)H\cdots O}$: Hydrogen bond length of the $O-H\cdots O$ HB, indicated by three dots.

$\angle O-H\cdots O$: Hydrogen bond angle of the $O-H\cdots O$ HB.

ΔR_{O-H}: Elongation of the proton-donor $O-H$ bond due to HB formation in cluster with respect to the corresponding monomer.

ν_{O-H}: Vibrational frequency (wave number) of the proton-donor $O-H$ stretching mode.

$\Delta\nu_{O-H}$: frequency shift of the proton-donor $O-H$ stretching mode as a result of HB formation.

$\Delta\nu_{O-H}$(SA) is calculated with respect to the ν_{OH}^{as}(SA) $= 3821 cm^{-1}$ which has stronger intensity.

Table S5: Relevant structural parameters related to hydrogen bond formation in (OA)(SA)$_2$ ternary clusters obtained at the MO6-2X/6-311++G(3df,3pd) level of calculation.

	PD*	R_{O-O} (Å)	$R_{(O)H\cdots O}$ (Å)	$\angle O-H\cdots O$ (degrees)	ΔR_{O-H} (Å)	ν_{O-H} (cm^{-1})	$\Delta\nu_{O-H}$ (cm^{-1})
(cTc)(SA)$_2$ −1	OA I	2.694	1.743	161.6	0.012	3454	-274
	SA I	2.641	1.647	175.7	0.029	3178	-643
(cTc)(SA)$_2$ −2	OA I	2.679	1.736	158.7	0.015	3501	-226
	OA II	2.679	1.736	158.7	0.015	3501	-226
	SA I	2.667	1.714	162.6	0.017	3417	-404
	SA II	2.667	1.714	162.6	0.017	3417	-404
(cTc)(SA)$_2$ −3	OA I	2.716	1.770	161.3	0.008	3581	-146
	SA I	2.571	1.574	171.4	0.038	3037	-784
(cTc)(SA)$_2$ −4	OA I	2.945	2.156	137.4	0.002	3701	-26
	OA II	2.667	1.715	160.7	0.017	3381	-346
	SA I	2.672	1.718	162.8	0.016	3536	-285
	SA II	2.694	1.795	150.4	0.016	3547	-274
(cTc)(SA)$_2$ −5	OA I	2.588	1.682	149.5	0.021	3277	-450
	SA I	2.920	2.115	139.3	0.005	3761	-60
	SA II	2.667	1.709	162.9	0.020	3413	-408
(cTt)(SA)$_2$ −1	OA I	2.625	1.631	173.9	0.030	3214	-605
	SA I	2.622	1.625	174.8	0.033	3091	-730
	SA II	2.706	1.745	163.5	0.021	3387	-434
(cTt)(SA)$_2$ −2	OA I	2.653	1.666	175.6	0.022	3376	-443
	OA I	2.517	1.491	152.1	0.062	2652	-1169
(cTt)(SA)$_2$ −3	OA I	2.616	1.690	154.1	0.023	3317	-456
	OA I	2.728	1.777	161.1	0.019	3435	-386
	SA II	2.767	1.865	152.7	0.008	3696	-125
(cTt)(SA)$_2$ −4	OA I	2.850	1.920	158.7	0.005	3684	-89
	OA II	2.740	1.740	165.8	0.017	3467	-352
	SA I	2.427	1.354	169.9	0.114	2010	-1811
(cTt)(SA)$_2$ −5	OA I	2.690	1.987	126.9	0.008	3596s, 3639as	-177,-134
	OA II	2.640	1.649	173.7	0.024	3133s, 3271as	-686,-548
	SA I	2.774	1.879	150.9	0.011	3596s, 3639as	-225,-182
	SA II	2.627	1.631	174.5	0.032	3133s, 3271as	-688,-550
(tTt)(SA)$_2$ −1	OA I	2.597	1.594	176.1	0.039	3260	-572
	OA II	2.597	1.594	176.1	0.039	3260	-572
	SA I	2.647	1.658	174.3	0.027	3012	-809
	SA II	2.647	1.658	174.3	0.027	3012	-809
(tTt)(SA)$_2$ −2	OA I	2.616	1.616	173.5	0.038	3032	-800
	OA I	2.706	1.764	159.2	0.017	3496s, 3446as	-336,-386
	SA II	2.711	1.817	149.2	0.020	3496s, 3446as	-336,-386
(tTt)(SA)$_2$ −3	OA I	2.665	1.680	174.4	0.021	3407	-424
	OA I	2.489	1.449	176.8	0.075	2451	-1370
(tTt)(SA)$_2$ −4	OA I	2.641	1.649	174.0	0.029	3240	-592
	OA I	2.598	1.595	175.5	0.038	3029	-792
	SA II	2.722	1.764	163.2	0.019	3420	-401
(tTt)(SA)$_2$ −5	OA I	2.770	1.797	170.6	0.043	3491	-341
	OA I	2.561	1.555	174.6	0.016	2966	-855
(tCt)(SA)$_2$ −1	OA I	2.599	1.595	173.5	0.041	2991	-830
	OA I	2.671	1.716	161.5	0.022	3385	-436
	SA II	2.652	1.667	168.0	0.032	3157	-664
(tCt)(SA)$_2$ −2	OA I	2.645	1.655	174.3	0.027	3257	-564
	OA II	2.645	1.655	174.3	0.027	3257	-564

	SA I	2.591	1.586	176.7	0.040	2993	-828
	SA II	2.591	1.586	176.7	0.040	2993	-828
$(tCt)(SA)_2$ −3	OA I	2.686	1.703	172.1	0.021	3362	-459
	OA I	2.557	1.552	173.9	0.042	2952	-869
$(tCt)(SA)_2$ −4	OA I	2.662	1.677	174.3	0.021	3383	-438
	OA I	2.486	1.445	177.2	0.076	2430	-1391
$(tCt)(SA)_2$ −5	OA I	2.639	1.653	170.8	0.028	3264	-557
	OA I	2.585	1.626	158.9	0.036	3108	-713
(cCt)(SA) −1	OA I	2.547	1.556	165.9	0.044	2954	-870
	SA I	2.665	1.681	172.4	0.024	3305	-516
	SA II	2.642	1.654	168.5	0.035	3102	-719
(cCt)(SA) −2	OA I	2.645	1.659	172.1	0.026	3267	-557
	SA I	2.573	1.576	171.6	0.037	3597	-224
(cCt)(SA) −3	OA I	2.627	1.637	175.5	0.026	3287	-537
	SA I	2.501	1.468	176.9	0.068	2548	-1273
(cCt)(SA) −4	OA I	2.697	1.720	170.2	0.020	3393	-431
	OA II	2.898	2.005	151.9	0.006	3734	-96
	SA I	2.513	1.482	178.5	0.065	2611	-1210
(cCt)(SA) −5	OA I	2.638	1.643	178.9	0.030	3213	-611
	OA II	2.734	1.766	171.7	0.010	3631	-199
	SA I	2.542	1.521	175.8	0.057	2746	-1075
	SA II	2.875	2.069	140.0	0.006	3737	-84

*PD: the proton-donor monomer for hydrogen bond (HB) formation – OA: Oxalic Acid donating proton via its OH bond to one of the SA molecules; SA: Sulfuric acid donating proton via its OH bond to OA. Additional labelling "I" and "II" have been used to distinguish between multiple proton donor groups of OA and SA monomers, as illustrated in Figure S1.

$R_{\mathrm{O-O}}$: Distance between the oxygen atom of OA and that of SA forming the $\mathrm{O-H\cdots O}$ HB.

$R_{\mathrm{(O)H\cdots O}}$: Hydrogen bond length of the $\mathrm{O-H\cdots O}$ HB, indicated by three dots.

$\angle\mathrm{O-H\cdots O}$: Hydrogen bond angle of the $\mathrm{O-H\cdots O}$ HB.

$\Delta R_{\mathrm{O-H}}$: Elongation of the proton-donor $\mathrm{O-H}$ bond due to HB formation in cluster with respect to the corresponding monomer.

$\nu_{\mathrm{O-H}}$: Vibrational frequency (wave number) of the proton-donor $\mathrm{O-H}$ stretching mode.

$\Delta\nu_{\mathrm{O-H}}$: frequency shift of the proton-donor $\mathrm{O-H}$ stretching mode as a result of HB formation.

$\Delta\nu_{\mathrm{O-H}}$(SA) is calculated with respect to the ν_{OH}^{as}(SA) $= 3821\mathrm{cm}^{-1}$ which has stronger intensity.

Figure S1: Geometries of the (OA)(SA)$_2$ ternary clusters obtained at the MO6-2X/6-311++G(3df,3pd) level of calculation.

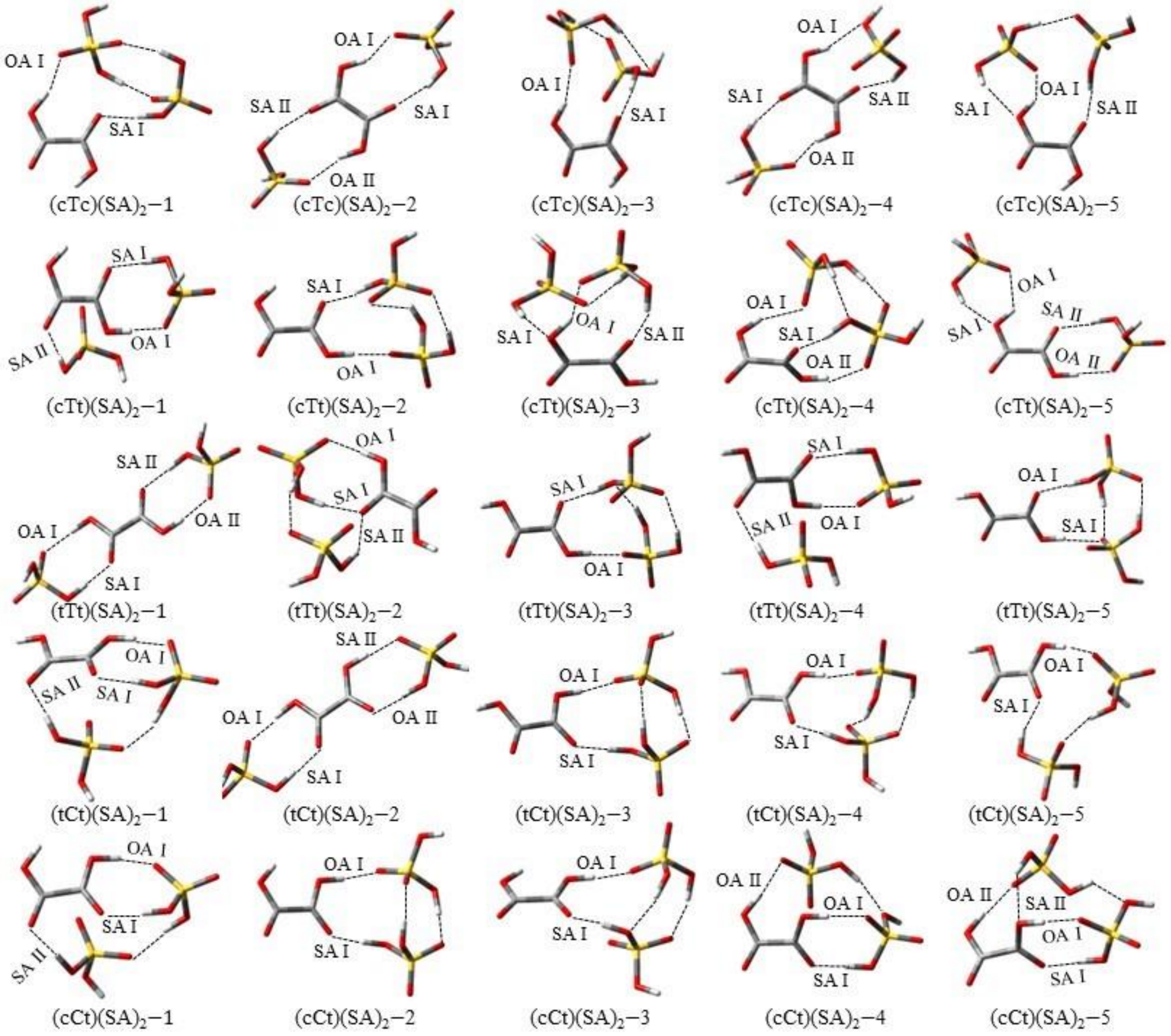

Table S5: Successive binding free energies (ΔG_S) for the formation of various (OA)(SA)$_2$ ternary clusters, derived from the addition of an SA monomer to the pre-existing (OA)(SA) binary clusters.

Final Channel	Initial channel	ΔG (kcal/mol)	Final Channel	Initial channel	ΔG (kcal/mol)
(cTc)(SA)$_2$−1	(cTc)(SA)−1 + SA	-6.27	(cTt)(SA)$_2$−1	(cTt)(SA)−1 + SA	-1.71
	(cTc)(SA)−2 + SA	-9.07		(cTt)(SA)−2 + SA	-5.17
	(cTc)(SA)−3 + SA	-11.32		(cTt)(SA)−3 + SA	-7.25
(cTc)(SA)$_2$−2	(cTc)(SA)−1 + SA	-4.28	(cTt)(SA)$_2$−2	(cTt)(SA)−1 + SA	-1.36
	(cTc)(SA)−2 + SA	-7.08		(cTt)(SA)−2 + SA	-4.81
	(cTc)(SA)−3 + SA	-9.34		(cTt)(SA)−3 + SA	-6.90
(cTc)(SA)$_2$−3	(cTc)(SA)−1 + SA	-0.82	(cTt)(SA)$_2$−3	(cTt)(SA)−1 + SA	0.04
	(cTc)(SA)−2 + SA	-3.62		(cTt)(SA)−2 + SA	-3.42
	(cTc)(SA)−3 + SA	-5.87		(cTt)(SA)−3 + SA	-5.51
(cTc)(SA)$_2$−4	(cTc)(SA)−1 + SA	0.47	(cTt)(SA)$_2$−4	(cTt)(SA)−1 + SA	0.02
	(cTc)(SA)−2 + SA	-2.34		(cTt)(SA)−2 + SA	-3.44
	(cTc)(SA)−3 + SA	-4.59		(cTt)(SA)−3 + SA	-5.52
(cTc)(SA)$_2$−5	(cTc)(SA)−1 + SA	3.06	(cTt)(SA)$_2$−5	(cTt)(SA)−1 + SA	1.30
	(cTc)(SA)−2 + SA	0.26		(cTt)(SA)−2 + SA	-2.16
	(cTc)(SA)−3 + SA	-1.99		(cTt)(SA)−3 + SA	-4.24
(tTt)(SA)$_2$−1	(tTt)(SA)−1 + SA	-5.13	(tCt)(SA)$_2$−1	(tCt)(SA)−1 + SA	-3.82
	(tTt)(SA)−2 + SA	-10.53		(tCt)(SA)−2 + SA	-9.67
	(tTt)(SA)−3 + SA	-11.56		(tCt)(SA)−3 + SA	-12.49
(tTt)(SA)$_2$−2	(tTt)(SA)−1 + SA	-1.01	(tCt)(SA)$_2$−2	(tCt)(SA)−1 + SA	-5.39
	(tTt)(SA)−2 + SA	-6.41		(tCt)(SA)−2 + SA	-11.24
	(tTt)(SA)−3 + SA	-7.44		(tCt)(SA)−3 + SA	-14.06
(tTt)(SA)$_2$−3	(tTt)(SA)−1 + SA	-1.87	(tCt)(SA)$_2$−3	(tCt)(SA)−1 + SA	-2.65
	(tTt)(SA)−2 + SA	-7.26		(tCt)(SA)−2 + SA	-8.51
	(tTt)(SA)−3 + SA	-8.30		(tCt)(SA)−3 + SA	-11.32
(tTt)(SA)$_2$−4	(tTt)(SA)−1 + SA	-0.42	(tCt)(SA)$_2$−4	(tCt)(SA)−1 + SA	-1.99
	(tTt)(SA)−2 + SA	-5.81		(tCt)(SA)−2 + SA	-7.84
	(tTt)(SA)−3 + SA	-6.85		(tCt)(SA)−3 + SA	-10.66
(tTt)(SA)$_2$−5	(tTt)(SA)−1 + SA	-0.50	(tCt)(SA)$_2$−5	(tCt)(SA)−1 + SA	1.03
	(tTt)(SA)−2 + SA	-5.89		(tCt)(SA)−2 + SA	-4.82
	(tTt)(SA)−3 + SA	-6.93		(tCt)(SA)−3 + SA	-7.63
(cCt)(SA)$_2$−1	(cCt)(SA)−1 + SA	-6.26			
	(cCt)(SA)−2 + SA	-16.25			
	(cCt)(SA)−3 + SA	-13.44			
(cCt)(SA)$_2$−2	(cCt)(SA)−1 + SA	-3.12			
	(cCt)(SA)−2 + SA	-13.10			
	(cCt)(SA)−3 + SA	-10.29			
(cCt)(SA)$_2$−3	(cCt)(SA)−1 + SA	-2.20			
	(cCt)(SA)−2 + SA	-12.19			
	(cCt)(SA)−3 + SA	-9.38			
(cCt)(SA)$_2$−4	(cCt)(SA)−1 + SA	1.22			
	(cCt)(SA)−2 + SA	-8.77			
	(cCt)(SA)−3 + SA	-5.96			
(cCt)(SA)$_2$−5	(cCt)(SA)−1 + SA	2.47			
	(cCt)(SA)−2 + SA	-7.52			
	(cCt)(SA)−3 + SA	-4.71			

Table S6: Boltzman-averaged values of Rayleigh scattering intensities ($<\Re>$), in a.u., of the OA conformers and their binary and ternatry clusters with AM and SA.

System	$\Re$ (a.u.)
cTc	68189
(cTc)(AM)	133571
$(cTc)(AM)_2$	226604
(cTc)(SA)	261626
$(cTc)(SA)_2$	553347
cTt	67140
(cTt)(AM)	128106
$(cTt)(AM)_2$	209337
(cTt)(SA)	255091
$(cTt)(SA)_2$	521593
tTt	68103
(tTt)(AM)	130516
$(tTt)(AM)_2$	211700
(tTt)(SA)	257981
$(tTt)(SA)_2$	577597
tCt	68198
(tCt)(AM)	130950
$(tCt)(AM)_2$	212246
(tCt)(SA)	258540
$(tCt)(SA)_2$	573019
cCt	69983
(cCt)(AM)	129003
$(cCt)(AM)_2$	209536
(cCt)(SA)	254713
$(cCt)(SA)_2$	521897
